# Heterogeneity of Prognostic Studies of 24-Hour Blood Pressure Variability: Systematic Review and Meta-Analysis

**DOI:** 10.1371/journal.pone.0126375

**Published:** 2015-05-18

**Authors:** Kathryn S. Taylor, Carl J. Heneghan, Richard J. Stevens, Emily C. Adams, David Nunan, Alison Ward

**Affiliations:** 1 Nuffield Department of Primary Care Health Sciences, University of Oxford, Oxford, United Kingdom; 2 Oxford University Hospitals Trust, Oxford, United Kingdom; University of British Columbia, CANADA

## Abstract

In addition to mean blood pressure, blood pressure variability is hypothesized to have important prognostic value in evaluating cardiovascular risk. We aimed to assess the prognostic value of blood pressure variability within 24 hours. Using MEDLINE, EMBASE and Cochrane Library to April 2013, we conducted a systematic review of prospective studies of adults, with at least one year follow-up and any day, night or 24-hour blood pressure variability measure as a predictor of one or more of the following outcomes: all-cause mortality, cardiovascular mortality, all cardiovascular events, stroke and coronary heart disease. We examined how blood pressure variability is defined and how its prognostic use is reported. We analysed relative risks adjusted for covariates including the appropriate mean blood pressure and considered the potential for meta-analysis. Our analysis of methods included 24 studies and analysis of predictions included 16 studies. There were 36 different measures of blood pressure variability and 13 definitions of night- and day-time periods. Median follow-up was 5.5 years (interquartile range 4.2–7.0). Comparing measures of dispersion, coefficient of variation was less well researched than standard deviation. Night dipping based on percentage change was the most researched measure and the only measure for which data could be meaningfully pooled. Night dipping or lower night-time blood pressure was associated with lower risk of cardiovascular events. The interpretation and use in clinical practice of 24-hour blood pressure variability, as an important prognostic indicator of cardiovascular events, is hampered by insufficient evidence and divergent methodologies. We recommend greater standardisation of methods.

## Introduction

Hypertension typically accounts for up to 50% of an individual’s cardiovascular (CV) risk.[1] Traditionally, this risk has been attributed to mean blood pressure (BP) load. However, it is now thought this explanation is unable to fully account for the effect of rising BP level on CV risk and the inherent variability of an individual’s BP may also be a significant contributing factor.[2]

The variability of BP can be evaluated over a variety of different timescales, over a lifetime,[3] years,[4] different seasons,[5] month-to-month (visit-to-visit),[6] day-to-day,[7] within 24 hours,[8] and by different methods: beat-to-beat, measured intra-arterially[9] or non-invasively.[10] The aetiology, pathogenesis and prognosis, of BP variability over these different timescales and methods are likely to vary considerably.[11]

Recent attention has focused on the predictive value of visit-to-visit and day-to-day BP variability[11] and the subsequent risk of stroke[12] and mortality.[13] The treatment of normotensives with antihypertensive agents, which reduce BP variability, can reduce CV morbidity, further supporting the hypothesis that variability is a potentially modifiable risk factor, regardless of baseline BP.[14] In addition, recent economic analyses and National Institute for Clinical Excellence (NICE) recommendations for the systematic use of ambulatory BP monitoring, could potentially increase the emphasis on the predictive ability of BP variability within 24 hours.[15–16] In this paper, we use the term ‘24-hour BP variability’ to refer to BP variability within 24 hours based on ambulatory BP measurement during the day, night or over 24 hours.

The prognostic value of 24-hour BP variability has been reviewed previously by a systematic review that included night dipping measures [17] and a mini-review of other 24-hour measures of BP variability including standard deviation.[18] Our aim was to revisit, update and expand on these reviews, by systematic review, in investigating to what extent the existing literature establishes the value of 24-hour BP variability as a prognostic index.

## Methods

### Data Sources and Search Strategy

We searched MEDLINE (1946-April 2013), EMBASE (1980-April 2013) and the Cochrane Library (Issue 3, 2013) using a sensitive search strategy for prognostic studies.[19,20] Our search terms included combinations of terms relating to BP variability (e.g. “blood pressure variation” and “dipping”), time of day (e.g. “day” “nocturnal” and “diurnal”) and cardiovascular risk (e.g. “hypertension”, “risk” and “mortality”). Our full search strategy is given in S1 Appendix. One author carried out the initial screen of titles and abstracts for relevance. In updating this initial search, papers were screened independently by two authors who first considered abstracts and titles, and then full texts and reference lists. Disagreements were resolved by discussion and review with a third author. Several authors were contacted for further information.

All extracted data were verified and checked, discrepancies were discussed and agreements on values reached. The list of extracted variables is given in S2 Appendix.

### Study Selection

Our study protocol is given in S3 Appendix. We included papers describing randomized controlled trials and observational cohort prognostic studies of 24-hour BP variability with: an adult population (aged 18 years or over); follow-up of at least one year; systolic, diastolic or both ambulatory BP measurements; BP measured at night, during the day or spanning 24 hours; and, reporting one or more of the following outcomes: all-cause mortality, CV mortality, all fatal and non-fatal CV events, stroke and coronary heart disease. Several groups of papers with duplicate or overlapping patient populations were included because they reported different measures, outcomes or methods of analysis. Each group was counted as a single study.

### Quality Assessment

Methodological quality was assessed by individual paper. Two authors independently carried out the assessments and disagreements were resolved by discussion and review with a third author. There is no established quality checklist for prognostic studies,[21] so we based our evaluation of quality on a framework for appraisal of prognostic studies[22] used previously.[23] We considered six criteria: (1) all were recruited in the same setting (from a general population or from a clinic population but not both); (2) clinical and demographic characteristics were described; (3) relative risks were adjusted for covariates including appropriate mean BP; (4) follow-up length was sufficient for the clinical outcomes (mean or median follow-up was at least five years); (5) follow-up was complete (at least 80%); and (6) outcomes assessment was objective or independently adjudicated.

### Statistical Analysis

Statistical analyses were by individual study and were carried out using STATA, version 13 (StataCorp, College Station, TX). Data from all studies that satisfied the eligibility criteria for inclusion in the systematic review were included in our analysis of methods to understand how 24-hour BP is described. We classified the measures of variability within a three-component framework: (1) class of measurement (e.g. standard deviation), (2) type of BP measured (e.g. systolic) and (3) timing (e.g. day). We examined the definitions of night and day.

Only studies that provided relative risks adjusted for the appropriate mean BP were included in our analysis of relative risks. Relative risks expressed as continuous variables were scaled to a common basis: 5mmHg increase for all dispersion measures; 10% increase for night-day ratio; 1mmHg increase for night dipping expressed as day-night difference; 10mmHg increase for measures of morning surge. We evaluated the potential for data pooling using meta-analysis and, where appropriate, we investigated further by applying data synthesis based on the DerSimonian and Laird method, inputting beta coefficients and standard errors into a random effects model. From the Mantel-Haenszel model we assessed the I-squared statistic for heterogeneity.[24] I-squared over 50% was defined as high heterogeneity.

In the analysis of relative risks, studies were separated by the hypertensive status of their patient populations. We defined a population as hypertensive if at least 80% were reported as hypertensive. We compared the predictive power of corresponding systolic and diastolic measures based on the same populations where possible. Sensitivity analyses assessed the effect of statistical heterogeneity and, in the diastolic-systolic comparison, the effect of relative risks rescaled to 1 SD increase.

## Results

**Fig 1:**
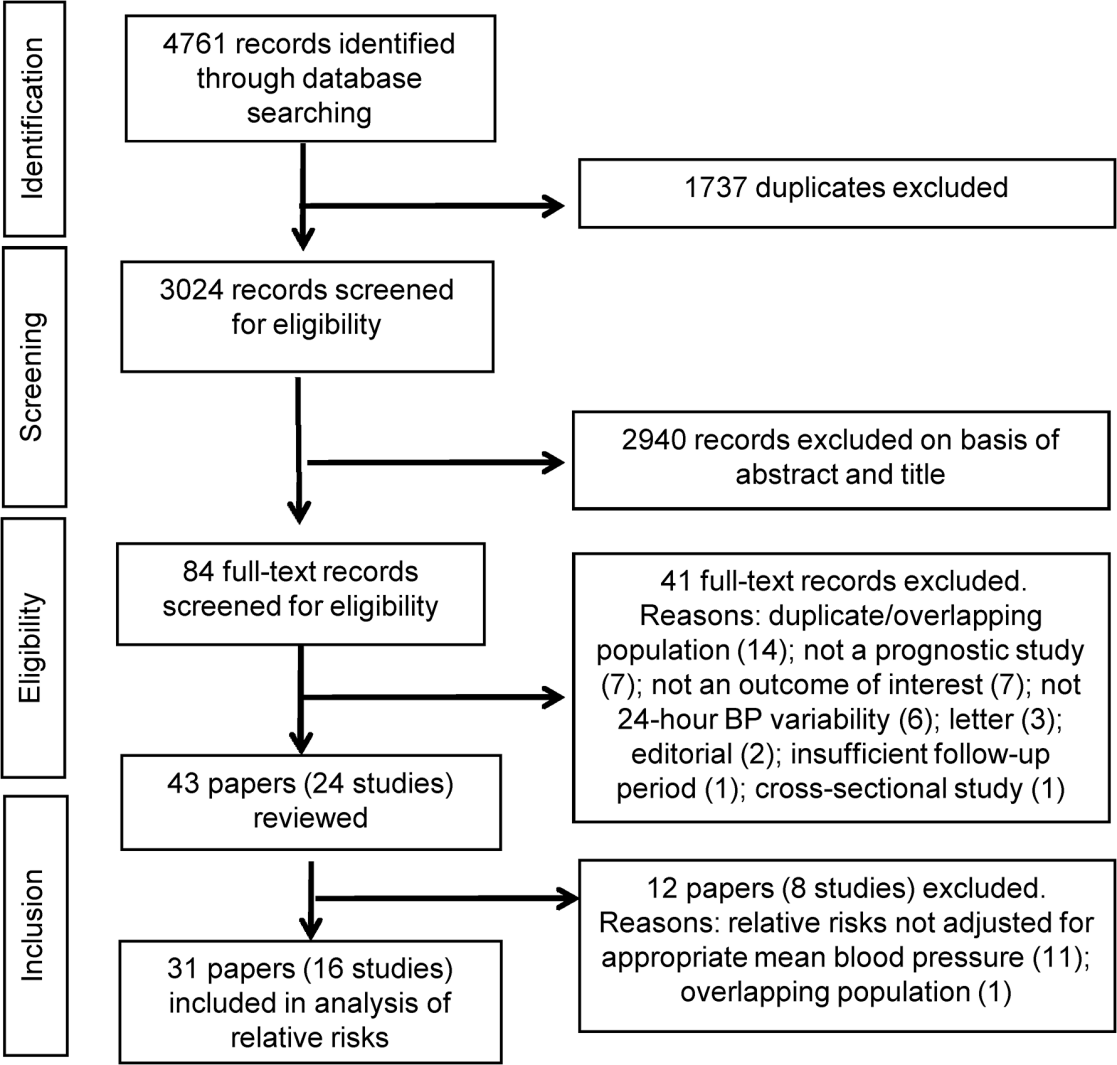
Study flow chart.

From 4,761 search results we screened 84 full-text records for eligibility (Fig 1) and, of these, 41 were excluded (S1 Table), leaving 43 included papers in our systematic review. These papers are listed in S2 Table where their references are numbered with a prefix “W”. Twenty-four papers were from five studies with overlapping or duplicate data sets: Chieti University (n = 3), International Database on Ambulatory Blood Pressure Monitoring in Relation to Cardiovascular Outcomes (IDACO, n = 10), which included several meta-analyses; Jichi Medical School (n = 4), Progetto Ipertensione Umbria Montioraggio Ambulatoriale (PIUMA, n = 5) and Hadassah Hebrew University (n = 2). There were 24 studies in total in our review based on the 43 papers (Table 1).

**Table 1:** Studies included in systematic review.

Ref	Study	Countries	Population	n	Age (yrs)	Follow-up (yrs)
**Chieti University**						
W1a	Pierdomenico 2005 *(excl)*	Italy	Hypertensive, untreated	1088	49.0	4.7
W1b	Pierdomenico 2006	Italy	Hypertensive, treated	1472	59.0^a^	4.9
W1c	Pierdomenico 2009	Italy	Hypertensive	1280	58.0^a^	4.8
**International Database on Ambulatory Blood Pressure Monitoring in Relation to Cardiovascular Outcomes (IDACO)**						
W2a	Boggia 2007*	Europe, Asia, South America	Mixed^b^	7458	56.8	9.6
W2b	Fagard 2008*	International	Hypertensive	3468	60.8	6.6
W2c	Fagard 2009*	International	Hypertensive	3468	60.8	6.6
W2d	Hansen 2006	Denmark	Mixed^b^	1700	55.8^a^	9.5
W2e	Hansen 2010*	Europe, Asia, South America	General	8938	53.0	11.3
W2f	Li 2010*	Europe, Asia, South America	General	5645	53.0	11.4
W2g	Metoki 2006	Japan	General	1430	61.1	10.4
W2h	Ohkubo 2002 *(excl)*	Japan	General	1542	61.0	9.2
W2i	Ohkubo 1997 *(excl)*	Japan	General	1542	61.0	5.1
W2j	Pringle 2003	Europe	Hypertensive	744	69.5	4.4
**Jichi Medical School**						
W3a	Eguchi 2009	Japan	Hypertensive, diabetes	300	67.8	4.5
W3b	Kabutoya 2010	Japan	Hypertensive	811	72.3	3.4
W3c	Kario 2001	Japan	Hypertensive	575	72.5^a^	3.4
W3d	Kario 2003	Japan	Hypertensive	519	72.0	3.4
**Progetto Ipertensione Umbria Monitoraggio Ambulatoriale (PIUMA)**						
W4a	Verdecchia1994	Italy	Hypertensive	959	52.9^a^	Not given
W4b	Verdecchia 1997	Italy	Hypertensive	1522	52.0	4.2
W4c	Verdecchia 2007	Italy	Hypertensive	2649	51.2	6.0
W4d	Verdecchia 2008 *(excl)*	Italy	Hypertensive	2934	50.9	7.0
W4e	Verdecchia 2012	Italy	Hypertensive	3012	50.8	8.4
**Hadassah Hebrew Univeristy**						
W5a	Ben Dov 2007	Israel	Mixed	3957	55.0	6.5
W5b	Israel 2011	Israel	Mixed	2627	56.5^a^	6.5
**Other studies**						
W6	Amici 2008	Italy	Mixed	42	65.9	5.0
W7	Astrup 2007 *(excl)*	Denmark	Type II diabetes mellitus	104	61.0^a^	9.2
W8	Bouhanick 2008 *(excl)*	France	Hypertensive, diabetes mellitus	97	60.8^a^	5.5
W9	Brotman 2008	US	Mixed	621	61.3	6.3^a^
W10	de la Sierra 2012	Spain	Hypertensive	2115	66.1	4.0
W11	Eto 2005	Japan	Hypertensive	106	73.9	2.8
W12	Gosse 2004	France	Hypertensive	507	49.0	7.0
W13	Iqbal 2011 *(excl)*	UK	Hypertensive	297	60.1	5.5
W14	Liu 2003 *(excl)*	Japan	End stage renal failure on haemodialysis	80	60.2^a^	2.8
W15	Mancia 2007	Italy	General	2012	50.9^a^	12.3
W16	Mena 2005	Venezuala	Mixed	312	66.9^a^	1.9
W17	Minutolo 2011	Italy, US	Hypertensive, non-dialysis chronic kidney disease	436	65.1	4.2
W18	Muxfeldt 2009	Brazil	Hypertensive	556	65.8	4.8
W19	Nakamura 1995 *(excl)*	Japan	Chronic ischaemic cerebro-vascular disease	76	63.7	2.3
W20	Nakano 2004	Japan	Type II diabetes mellitus	364	54.0	7.2
W21	Otsuka 1996 *(excl)*	Japan	Hypertensive	176	51.6^a^	6.0
W22	Rothwell 2010	Europe	Hypertensive	843	Not given	5.5
W23	Sturrock 2000 *(excl)*	UK	Diabetes mellitus	75	52.1^a^	3.5
W24	Zweiker 1994 *(excl)*	Austria	Hypertensive	116	59.0	2.6

Abbreviations: *(excl)*—Excluded from analysis of relative risks; ARV—average real variability; CoV—coefficient or variation; CV—cardiovascular; MS—morning surge; SD—standard deviation.

*Relative risks/hazard ratios based on meta-analysis

^a^ Given for subgroups of patients so mean for all patients was estimated by calculating a weighted average

^b^ Provided separate relative risks for hypertensive and normotensive patients for night-day ratio (Boggia 2007, n = 3436 hypertensive, n = 4022 normtensive, CV events and all-cause mortality, systolic BP; Hansen 2006, n = 682 hypertensive, n = 1018 normotensive, CV events, systolic and diastolic BP)

Of these 24 studies, 13 had populations from within Europe, nine had non-European populations and two studies were based on international databases. Thirteen studies involved hypertensive populations, of which two studies involved patients with diabetes and one study involved patients with chronic kidney disease. One study reported hypertensive and mixed populations. Ten studies involved populations with mixed hypertensive status. Of these, five were reported as general or mixed populations, one had a population with chronic ischemic cerebrovascular disease, three had a population with diabetes and another study had a population with end-stage renal failure. Numbers of patients enrolled in the studies varied between 42 and 8938 (median 843, interquartile range 300–2115). Average age of the patient population varied between 49.0 and 73.9 years (median 60.2, interquartile range 53.0–65.1 years).

### Methodological Quality Assessments

We assessed the methodological quality of 42 of the 43 papers as insufficient information was provided to evaluate the quality of one paper.(W22)

No papers met all six criteria, 19 papers met five criteria, 12 met four criteria, 10 met three criteria, and one paper met two criteria (S3 Table). The number of papers satisfying each criterion ranged from 12 to 40 papers. Length of follow-up varied between 1.9 years and 12.3 years (median 5.5, interquartile range 4.2 to 7.0 years), was missing in one paper (W4a) and was less than five years in 17 papers, with follow-up ranging from 1.9 to 4.9 years. The criterion most often met was providing a description of clinical and demographic characteristics (40 papers, 95.2%) and the criterion least often met was outcomes assessment being objective or independently adjudicated (12 papers, 28.6%).

### Describing 24-Hour BP Variability

Reviewing the literature, we found that 24-hour BP variability was referred to, in general terms, as “short-term variability” [25] or “24-hour ambulatory blood pressure variability” (W13), or more specifically as “diurnal” variation or variability (W9), “circadian” blood pressure profile, pattern, variation or variability [26] (W4b, W4d), or with reference to a particular type, or measure, of 24-hour BP variability.

### Measuring 24-Hour BP Variability

Among the 24 included studies, we found 36 different measures of 24-hour BP variability across the three different components of variation: class, type and timing (Table 2).

**Table 2:** Components of 24-hour BP variability measures.

Class	BP type	Timing	No of studies (references)	No of measures
**Dispersion measures**				
Standard deviation	Systolic or diastolic	Day, night or 24hrs	8 (W1a, W1b, W1c, W2e, W2j, W3a, W4c, W11, W15, W16, W21)	6
Coefficient of var.^a^	Systolic	Day or 24hrs	2 (W11,W22)	2
Average real var.^b^	Systolic or diastolic	24hrs	3 (W1c, W2e, W16)	2
SDday-night^c^	Systolic or diastolic	24hrs	1 (W2e)	2
Circadian amplitude	Systolic or diastolic	24hrs	1 (W21)	2
**Night dipping measures**				
Night dipping 1 (night-day ratio)^d^	Systolic, diastolic or both	24hrs	14 (W2a, W2b, W2c, W2d, W2g, W2h, W2i, W3a, W3b, W3c, W4a, W4b, W4d, W4e, W5a, W7, W8, W9, W10, W13, W14, W17, W18, W23, W24)	3
Night dipping 2^e^	Systolic, diastolic or both	24hrs	3 (W15, W19, W20)	3
Night dipping 3^f^	Systolic or diastolic	24hrs	1 (W21)	2
**Morning pressure surge measures**				
Preawakening 1^g^	Systolic	24hrs	4 (W2f, W2g, W3d, W4e, W5b)	1
Preawakening 2^h^	Systolic	24hrs	1 (W12)	1
Preawakening 3^i^	Systolic	24hrs	1 (W5b)	1
Preawakening 4^j^	Systolic	24hrs	1 (W5b)	1
Sleep trough 1^k^	Systolic or diastolic	24hrs	2 (W3a, W4e)	2
Sleep trough 2^l^	Systolic	Both	4 (W2f, W2g, W3d, W5b, W6)	1
Sleep trough 3^m^	Systolic	Both	1 (W5b)	1
Sleep trough 4^n^	Systolic	Both	1 (W13)	1

Based on 24 studies included in the review.

^a^standard deviation / mean BP

^b^ average of absolute value of successive pairs of BP measurements

^c^ weighted average of day standard deviation and night standard deviation

^d^percentage nocturnal fall

^e^ difference of mean day BP and mean night BP

^f^ adjusted day-night difference, (mean day BP-mean night BP)/mean 24-hour BP

^g^mean BP in 2 hours after awakening—mean BP in 2 hours preawakening

^h^difference between BP on rising and BP in 30 minutes before rising

^i^difference between mean BP in first hours after awakening and mean BP in last hour preawakening

^j^difference between mean BP in 3 hours after awakening and mean BP in 3 hours preawakening

^k^difference between mean BP in 2 hours after awakening and mean of the 3 BP readings centred on the lowest BP readings during sleep

^l^difference between mean BP in 2 hours after awakening and mean of all the BP readings during sleep

^m^difference between mean BP in 2 hours after awakening and lowest mean of 3 BP consecutive BP readings during the night

^n^≥20/15mmHg rise in first two BP readings from 7am compared to average night BP

#### Class of measurement

We identified 3 measures of night dipping, 8 measures of morning pressure surge and 5 measures of dispersion. Among nine studies reporting dispersion measures, standard deviation (SD) was the dispersion measure most often reported (n = 8). Eighteen studies reported measures of night dipping which differed in their unit of measurement (Table 2). Most (n = 14) reported dipping measures defined by percentage fall or an equivalent definition based on night-day ratio. Seven studies reported morning surge measures. The morning surge classes differed in the timing and number of BP measurements used in the definition of the BP surge. There were nine different definitions of morning surge.

#### Type of BP measured

Of 36 measures, 21 (58.3%) measures were based on readings of systolic BP only, 12 (33.3%) on diastolic BP only, and three (0.8%) were based on both BPs, either monitored together (W2d, W2h, W2i, W4a) or combined in a weighted average (W7, W19).

#### Timing of measurement

Of 36 BP variability measures, seven were based on day-time readings only or night-time readings only (Table 2). Of 24 studies, definitions of day-time and night-time were given by 21 studies, producing 13 different definitions (Table 3). The most common definition was based on the time spent in and out of bed (n = 8).

**Table 3:** Variation of definitions of night and day.

Daytime	Day-time hours	Night-time	Night-time hours	No of studies	References
**Diary entries**					
Time awake or time between rising and lying down	variable	Time asleep or time between lying down and rising	variable	8	W1a, W1b, W1c, W3a, W3b, W3c, W3d, W4b, W4d, W4e, W5a, W5b, W10, W12, W17, W18
Time when > 8 hours awake	variable	Time when > 4 hours asleep	variable	1	W2g, W2h, W2i
**Short fixed clock-time intervals**					
10am to 8pm	10	Midnight to 6am	6	3	W2a, W2b, W2c, W2e, W2f, W2j, W4c, W4d, W21
9am to 9pm	12	1am to 6am	5	1	W22
10am to 10.59pm	13	1am to 6.59pm	6	1	W23
**Long fixed clock-time intervals**					
6am to 10pm	16	10pm to 6am	8	3	W4a, W4d, W19, W20
7am to 11pm	16	11pm to 7am	8	1	W15
6am to midnight	18	Midnight to 6am	6	2	W2d, W18
7am to 10pm	15	10pm to 7am	9	1	W8
9am to 9pm	12	9pm to 9am	12	1	W24
8am to 8pm	12	8pm to 8am	12	1	W14
6am to 9pm	15	9.30pm to 5.30am	8	1	W11
6am to 11pm	17	11pm to 6am	7	2	W9, W16

In studies with multinational patient populations, adjustments were made for time differences. Three studies did not provide definitions.(W6, W7, W13)

### Defining Cardiovascular Outcomes

The most commonly studied outcomes were all fatal and non-fatal CV events were the most common (18 studies of each, 75.0%) and coronary heart disease was the outcome least often reported (2 studies). Authors used 26 different terms to refer to cardiovascular events. These definitions involved between two and 11 conditions (median 5 conditions, inter-quartile range 4–7). Myocardial infarction and stroke were the conditions most often included in definitions. Twelve studies defined stroke of which four included transient ischemic attack in the definition. Two studies defined coronary heart disease, each providing a different definition.

### Predicting Cardiovascular Outcomes

Fourteen studies (27 papers) and 25 of the 31 measures of BP variability were included in our analysis of relative risks (Fig 1).

#### Expressing relative risk

Associations of BP variability with cardiovascular outcomes were expressed as categorical or continuous expressions of relative risks or hazard ratios. Categorisations included: for dispersion measures, ‘high’ and ‘low’, defined by above vs. below the mean (W11); for night dipping measures, with varying thresholds, two, three of four categories, risers (also known as reverted or inverted dippers), non-dippers (reduced or decreased dippers), dippers (normal dippers) and extreme dippers; and, for morning surge measures, two or more categories with thresholds based on the top decile,(W2f, W3d) top tertile,(W6) quintiles(W2g, W18) or quartiles(W4e) of the morning pressure surge. Of 24 papers reporting relative risks expressed for categories of night dipping, four (16.7%) also reported relative risks expressed as a continuous variable in the same paper.(W2a, W2d, W10, W18)

Of 43 papers, 32 (74.4%) adjusted for covariates including the appropriate mean BP, 5 (11.6%) adjusted for covariates excluding the appropriate mean BP and 6 papers (14.0%) provided unadjusted analyses.

#### Extent of study of measures

**Fig 2:**
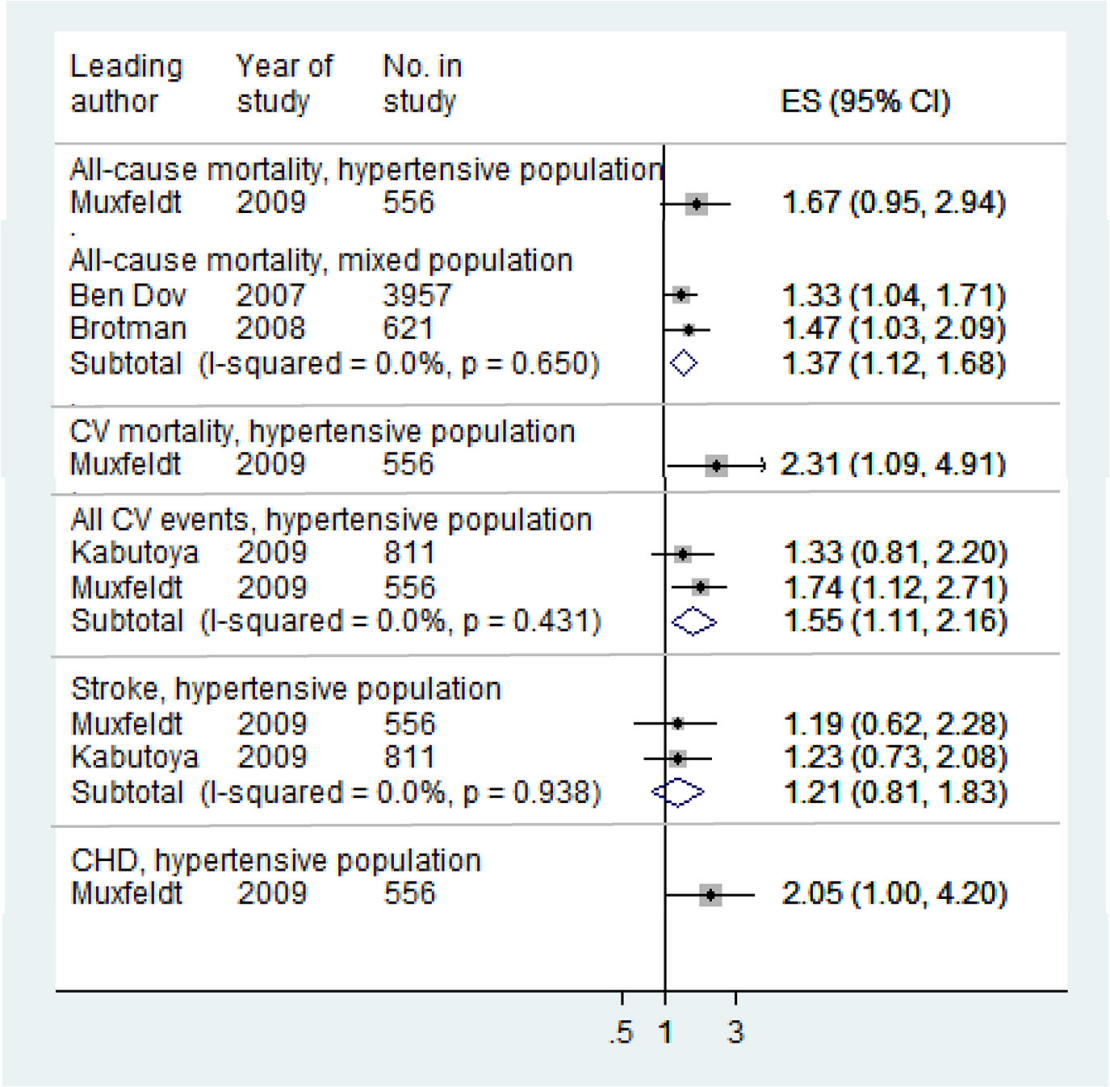
Associations of systolic night dipping 1 with cardiovascular outcomes: non-dippers verses dippers for all patients. Relative risks:>1 increased risk;< 1 reduced risk.

**Fig 3:**
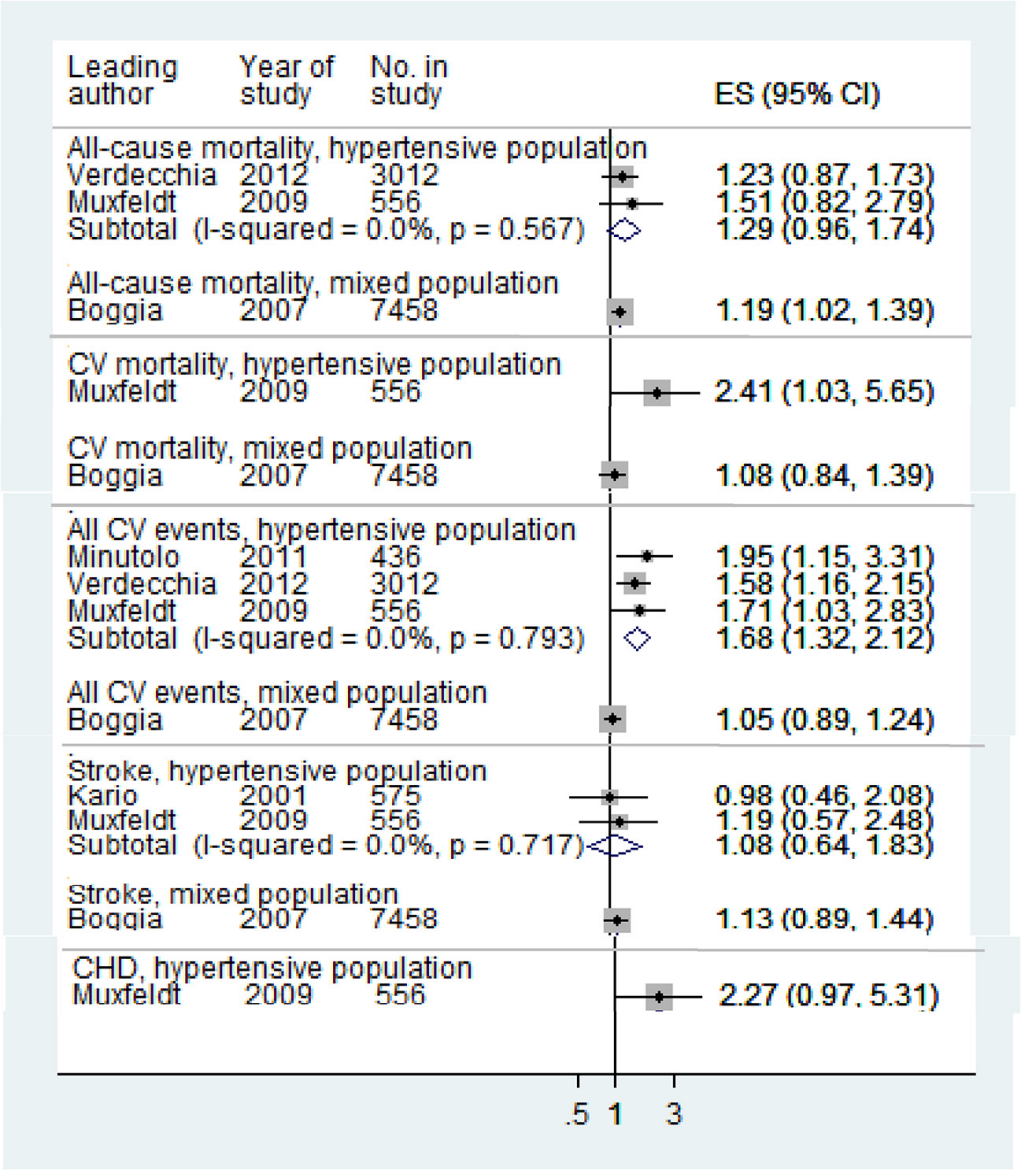
Associations of systolic night dipping 1 with cardiovascular outcomes: non-dippers verses dippers, excluding risers and dippers. Relative risks:>1 increased risk;< 1 reduced risk. Pooled relative risk for all CV events, hypertensive populations, excluding Minutolo 2009 (non-dialysis chronic kidney disease): 1.61 (1.24, 2.10)

**Fig 4:**
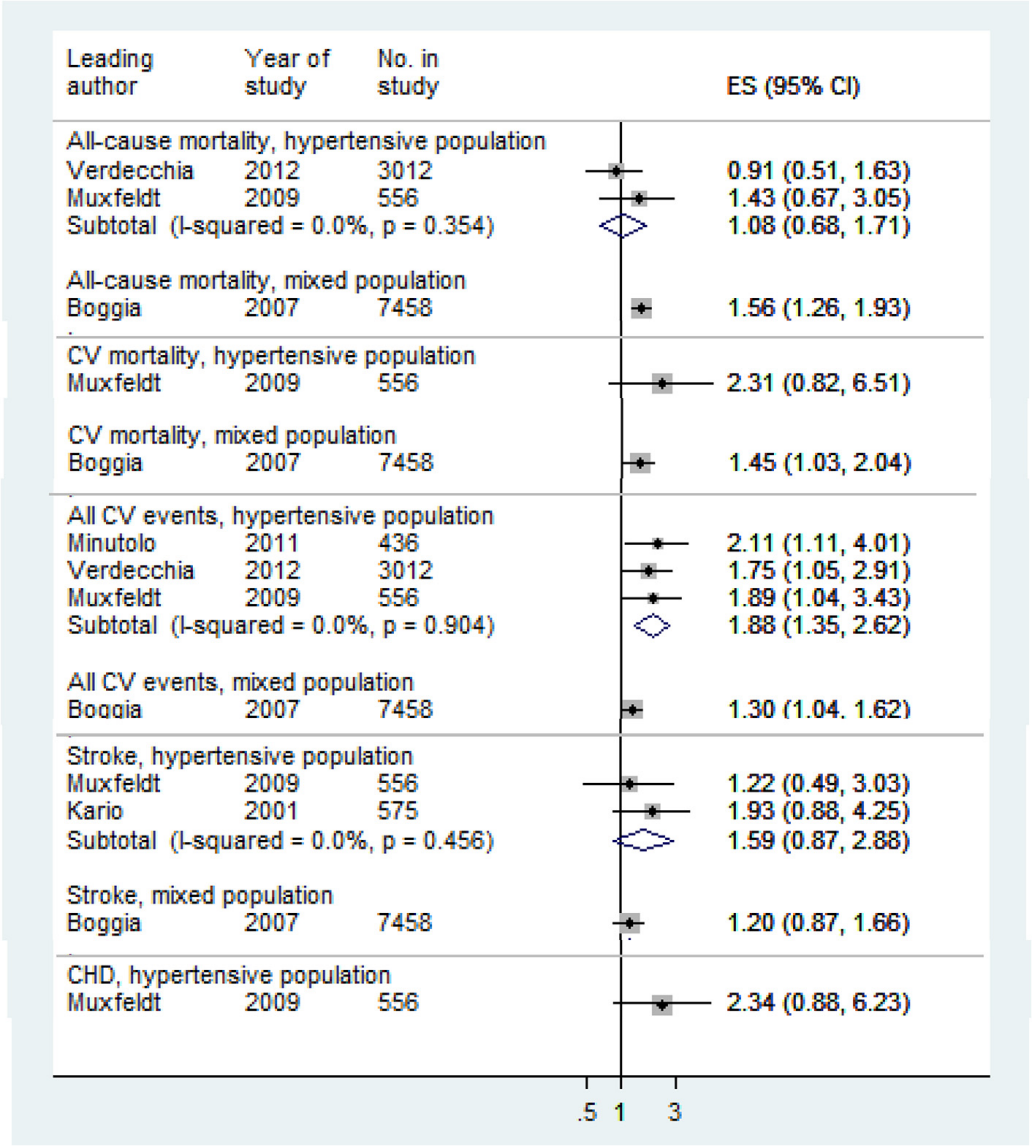
Associations of systolic night dipping 1 with cardiovascular outcomes: risers verses dippers. Relative risks:>1 increased risk;< 1 reduced risk. Pooled relative risk for all CV events, hypertensive populations, excluding Minutolo 2009 (non-dialysis chronic kidney disease): 1.81 (1.23, 2.66)

**Fig 5:**
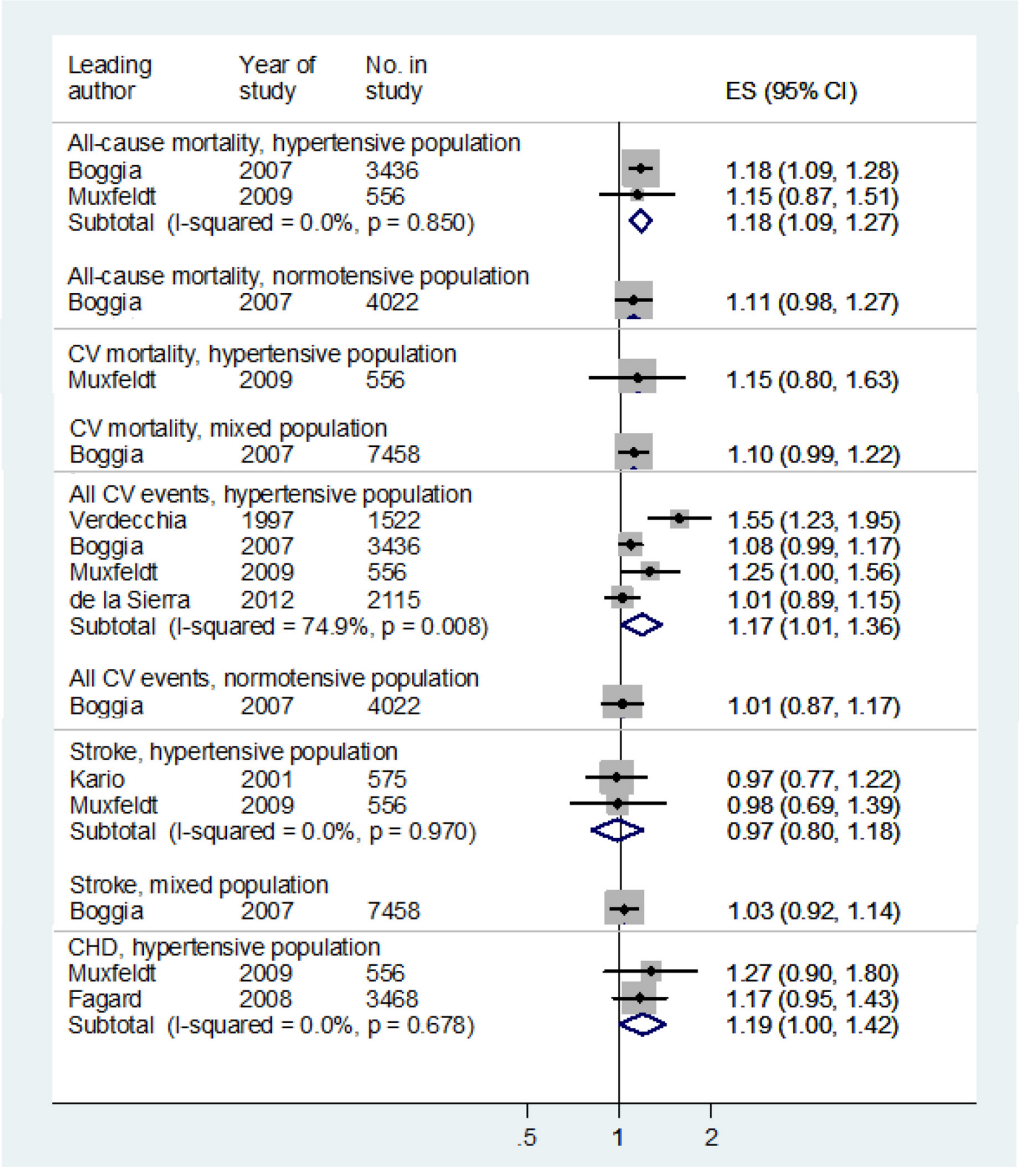
Associations of night-day ratio with cardiovascular outcomes. Relative risks:>1 increased risk;< 1 reduced risk. Pooled relative risk for all CV events, hypertensive population excluding de la Sierra 2012 only (did not control for treatment): 1.25 (1.00, 1.56), I-squared = 79.0%; excluding Verdecchia 1997 only:1.07 (0.99, 1.17), I-squared = 23.7%; excluding de la Sierra 2012 and Verdecchia 1997: 1.12 (0.98, 1.27), I-squared 33.6%.

Night dipping, based on percentage change (Night dipping 1) was the only measure for which relative risks were reported by more than two studies, and therefore the only measure for which we pooled relative risks (Table 4 and S4 Table). For both hypertensive and mixed populations, night dipping was associated with lower risk of cardiovascular events (Figs 2 and 3) while rising blood pressure at night compared to day was associated with increased risk (Fig 4). Night-day ratio (Fig 5) also provided evidence of predictive power in hypertensive populations, as did dispersion measures in general populations (Table 5).

**Table 4:** Numbers of studies reporting 24-hour blood pressure variability measures as a prognostic index of cardiovascular events.

Class	BP	Timing	All-cause mortality	CV mortality	All CV events	Stroke	CHD
**Hypertensive populations**							
SD	Systolic	Day	0	1	2	1	0
SD	Systolic	Night	0	1	2	1	0
SD	Diastolic	Day	0	0	1	0	0
SD	Diastolic	Night	0	0	1	0	0
CoV	Systolic	Day	0	0	1	0	0
Night dipping 1	Systolic	24 hrs	2*	1	4*	2	2*
Night dipping 1	Diastolic	24 hrs	1*	1	1*	1	2*
Pre-awakening 1	Systolic	24 hrs	0	0	1	1	0
Pre-awakening 2	Systolic	24 hrs	0	0	1	0	0
Sleep trough 1	Systolic	24 hrs	0	0	2	0	0
Sleep trough 1	Diastolic	24 hrs	0	0	1	0	0
Sleep trough 2	Systolic	24 hrs	0	0	0	1	0
**Mixed populations**							
SD	Systolic	Day	1	1	0	0	1
SD	Systolic	Night	1	1	0	0	0
SD	Systolic	24 hrs	2*	2*	1* †	1*	0
SD	Diastolic	Day	1	1	0	0	0
SD	Diastolic	Night	1	1	0	0	0
SD	Diastolic	24 hrs	2*	2*	1*	1*	0
ARV	Systolic	24 hrs	1*	1*	1*	1*	0
ARV	Diastolic	24 hrs	1*	1*	1*	1*	0
SDday-night	Systolic	24 hrs	1*	1*	1*	1*	0
SDday-night	Diastolic	24 hrs	1*	1*	1*	1*	0
Night dipping 1‡	Systolic	24 hrs	1*	1*	1*	1*	0
Night dipping 1‡	Diastolic	24 hrs	1*	1*	1*	1*	0
Night dipping 2	Systolic	24 hrs	1	1	0	0	0
Night dipping 2	Diastolic	24 hrs	1	1	0	0	0
Pre-awakening 1	Systolic	24 hrs	1	0	0	1	0
Pre-awakening 3	Systolic	24 hrs	1	0	0	0	0
Pre-awakening 4	Systolic	24 hrs	1	0	0	0	0
Sleep trough 2	Systolic	24 hrs	1	0	0	0	0
Sleep though 3	Systolic	24 hrs	1	0	0	0	0

Providing continuous expressions of relative risk.

* Includes studies reporting relative risks/hazard ratios based on pooled data from other studies.

†Further data from an extra study could not be included as there was insufficient information to extract the data

‡Night-day ratio; SD—standard deviation, ARV—average real variability, CoV—coefficient of variation

**Table 5:** Comparing predictive power of corresponding systolic and diastolic measures.

							Systolic			Diastolic		
No	Author	n	Population	BPV measure	BP	Outcome	RR	95% CI		RR	95% CI	
1	Hansen 2010	8938	G	SD, 24hrs	Systolic	All cause mortality	1.00	0.98	1.02	1.04	1.01	1.06
2	Mancia 2007	2012	G	SD, 24hrs	Systolic	All cause mortality	1.00	0.84	1.20	1.19	0.94	1.51
3	Mancia 2007	2012	G	SD, day	Systolic	All cause mortality	1.17	0.99	1.38	1.41	1.14	1.75
4	Mancia 2007	2012	G	SD, night	Systolic	All cause mortality	1.09	0.91	1.32	1.26	1.01	1.58
5	Hansen 2010	8938	G	SDdn	Systolic	All cause mortality	1.03	1.00	1.06	1.08	1.05	1.12
6	Hansen 2010	8938	G	ARV	Systolic	All cause mortality	1.05	1.02	1.08	1.07	1.04	1.11
7	Boggia 2007	7458	M	Night dipping 1	Systolic	All cause mortality	1.17	1.09	1.25	1.13	1.06	1.21
8	Muxfeldt 2009	556	H	Night dipping 1	Systolic	All cause mortality	1.15	0.87	1.51	0.98	0.77	1.25
9	Mancia 2007	2012	G	Night dipping 2	Systolic	All cause mortality	0.99	0.97	1.00	0.98	0.96	1.00
10	Hansen 2010	8938	G	SD, 24hrs	Systolic	CV mortality	1.01	0.98	1.04	1.06	1.02	1.10
11	Mancia 2007	2012	G	SD, 24hrs	Systolic	CV mortality	0.91	0.66	1.26	1.15	0.76	1.76
12	Mancia 2007	2012	G	SD, day	Systolic	CV mortality	1.21	0.89	1.66	1.67	1.13	2.48
13	Mancia 2007	2012	G	SD, night	Systolic	CV mortality	1.05	0.76	1.45	1.29	0.86	1.93
14	Hansen 2010	8938	G	SDdn	Systolic	CV mortality	1.02	0.98	1.06	1.10	1.04	1.15
15	Hansen 2010	8938	G	ARV	Systolic	CV mortality	1.07	1.03	1.12	1.12	1.07	1.17
16	Boggia 2007	7458	M	Night dipping 1	Systolic	CV mortality	1.10	0.99	1.22	1.11	1.00	1.24
17	Muxfeldt 2009	556	H	Night dipping 1	Systolic	CV mortality	1.15	0.80	1.63	1.02	0.76	1.38
18	Mancia 2007	2012	G	Night dipping 2	Systolic	CV mortality	0.98	0.95	1.00	0.96	0.93	0.99
19	Hansen 2010	8938	G	SD, 24hrs	Systolic	CV events	1.01	0.99	1.03	1.02	0.99	1.05
20	Eguchi 2009	300	HD	SD, day	Systolic	CV events	1.15	0.82	1.58	2.03	0.88	1.35
21	Eguchi 2009	300	HD	SD, night	Systolic	CV events	1.38	1.00	1.92	1.85	1.19	2.89
22	Hansen 2010	8938	G	SDdn	Systolic	CV events	1.02	0.99	1.04	1.04	1.00	1.07
23	Hansen 2010	8938	G	ARV	Systolic	CV events	1.03	1.00	1.06	1.04	1.01	1.08
24	Pierdomenico 2009	1280	H	ARV	Systolic	CV events	2.07	1.31	3.28	1.36	0.92	2.02
25	Boggia 2007	7458	M	Night dipping 1	Systolic	CV events	1.06	0.98	1.15	1.08	1.01	1.15
26	Muxfeldt 2009	556	H	Night dipping 1	Systolic	CV events	1.25	1.00	1.56	1.13	0.94	1.37
27	Eguchi 2009	300	HD	Sleep trough 1	Systolic	CV events	1.02	0.86	1.24	1.05	0.83	1.36
28	Hansen 2010	8938	G	SD, 24hrs	Systolic	Stroke	0.99	0.96	1.03	1.03	0.89	1.20
29	Hansen 2010	8938	G	SDdn	Systolic	Stroke	1.01	0.97	1.06	1.05	0.99	1.11
30	Hansen 2010	8938	G	ARV	Systolic	Stroke	1.04	1.00	1.09	1.08	1.03	1.13
31	Boggia 2007	7458	M	Night dipping 1	Systolic	Stroke	1.03	0.92	1.14	1.04	0.94	1.16
32	Muxfeldt 2009	556	H	Night dipping 1	Systolic	Stroke	0.98	0.69	1.39	0.96	0.72	1.28
33	Muxfeldt 2009	556	H	Night dipping 1	Systolic	CHD	1.27	0.90	1.80	1.11	0.83	1.48

G—general; M—mixed; H—hypertensive; HD—hypertensive, diabetes; SD—standard deviation; ARV—average real variability; RR—relative risk. Relative risks were scaled to the same increase except Pierdomenico 2009 (categorical expressions of relative risks). Sensitivity analysis (relative risks rescaled to 1 SD increase) are shown in S5 Table.

Other variability measures for which the predictive value for different cardiovascular outcomes has been assessed by more than one study are the diastolic and systolic measures of standard deviation (Table 4), again largely based on studies of patients with mixed hypertensive status. Further measures which have been researched by single studies across different cardiovascular outcomes are the systolic measures of pre-awakening 1 and sleep trough 2, which were evaluated over mixed populations (S4 Table). Measures, which have been evaluated less well include coefficient of variation, most of the morning surge measures and diastolic measures in general (Table 4).

#### Comparing the predictive power of systolic and diastolic measures

Comparisons could be made using data from five papers (four studies). When relative risks were scaled to the same increase, involving 33 comparisons (Table 5), the diastolic measure had stronger predictive power than the systolic measure in 15 cases (45.5%), weaker power in 3 cases (9.1%) and neither had predictive power in 15 cases (45.5%). When relative risks were scaled to 1 SD increase, involving 30 comparisons (S5 Table), the diastolic measure had stronger predictive power in 14 cases (46.7%), weaker power in 7 cases (10.0%) and neither had predictive power in 13 cases (43.3%). The majority of these comparisons involved general populations.

## Discussion

In our review, we found variation of terms used to refer to blood pressure variability within 24 hours and a diverse set of measures of 24-hour BP variability with variation in the definitions and terminology of individual measures. Several measures of BP variability depended on differences between day-time BP and night-time BP but there were no universally consistent definitions of the hours that constitute day and night. Further inconsistencies exist in the definitions of CV outcomes and in the expression of relative risks.

The power of prediction varied across the variability measures well researched and other measures less well-researched. The basis of analysis varied widely, between one and five studies and involving between less than 100 patients and several thousand patients. We have reported the smaller studies to highlight the diversity of methods.

Night dipping, or lower night-time blood pressure, based on percentage change was the most researched measure, and having relative risks reported by more than two studies, across cardiovascular outcomes. Night dipping was associated with lower risk of cardiovascular events. In many cases the predictive power of measures had only been assessed by a single study

The majority of 24-hour BP variability measures involved systolic BP measurements. Where we could compare the prognostic value of systolic and diastolic measures, we found measures of diastolic BP variability had higher predictive power more often than not compared to the corresponding systolic measures.

Our findings are consistent with those of other studies where variations in methods and definitions were reported[17, 27–29] The superiority of diastolic BP variability measures over systolic measures has also been reported. (W2e, W15) It has been suggested that the differences are accounted for by arterial stiffness(W2e) and the greater dependence of overall BP variability on diastolic BP as it covers a greater proportion of the cardiac cycle.(W15)

We have presented a comprehensive, systematic review of the methods of analysis and prognostic value of 24-hour BP variability. We expand on previous reviews[17,18] by exploring methods of analysis further, by considering other measures of BP variability and examining relative risks of more cardiovascular outcomes, and via systematic review. Our study is also strengthened by narrowing our inclusion criteria to prospective cohort studies and populations from randomised controlled trials. By reviewing the literature systematically, we avoided selection bias. Restricting our analysis to fully-adjusted relative risks reduced the risk of confounding.

There were twice as many studies of hypertensive populations than general populations in our review which suggests possible selection bias. We could not account for possible bias. Nor could we account for physical activity during ambulatory monitoring or address the methodological quality limitations, nor the significance of the variation of definitions of cardiovascular events and day-time and night-time definitions of the studies in our review. Our analysis of the predictive value of measures is tentative, given the heterogeneity of studies.

It is understood that the prognostic significance of diastolic BP declines with age while that of systolic BP increases with age[30] but, given the limitations of our data, we were unable to investigate this issue further. A more comprehensive assessment would compare the predictions of systolic and diastolic BP of more measures, across different populations, involve more studies, and stratify by age, across different cardiovascular outcomes.

Whilst new measures of 24-hour BP variability have been proposed,[31–34] there is clearly a need to focus on understanding better the predictive value of fewer measures and a better standardisation of methods. Concerns have been expressed about categorizations of patients by night dipping status being mainly based on arbitrary criteria and the inability to reproduce as patients can change dipping status between readings.[17,35,36] There are several other concerns about categorising continuous variables including loss of statistical power, obscuring any non-linearity in the relation between variable and outcome, and the problem with viewing those with similar values but on opposite sides of the threshold very differently.[37] It has been recommended that, for BP variability measures of nocturnal fall, categorical expressions of relative risks are accompanied by continuous analyses and that relative risks, based on ambulatory BP data, are adjusted for the appropriate mean BP.[17](W2a)

There are no guidelines on how day-time and night-time should be measured. [38] The most common definitions of night-time and day-time were based on time asleep and awake but measuring these accurately is challenging, as highlighted by others [17]. Patient-filled diary cards are simple and popular [39] but can be inaccurate due to recall bias. Accelerometry-based monitors could provide more accurate estimations of periods asleep and awake,[40] but these devices are not widely available. The use of narrow, fixed clock-time intervals have defined day-time and night-time BPs that are approximately within 1–2mmHg of the BPs when awake and asleep.[41] This is achieved by avoiding the transition periods in the morning and evening when rapid changes in BP can occur[41] and involve the periods when a variable proportion of patients may be in or out of bed.(W4d) Consistency in the prognostic value of diurnal BP variability across these different definitions has been reported, (W4d) which may suggest that variations in definitions of night and day are less important than other sources of variation in methods.

The existence of methodological and clinical heterogeneity[42] leads to substantial difficulties in interpreting prognostic data. The sheer number of measures limits the utility of 24-hour BP variability and its interpretation and use in clinical practice, particularly as an important prognostic indicator of CV events. If variability is going to inform clinical practice, there is an urgent need to harmonize methodology sufficiently to draw conclusions across the research field with emphasis of future research directed towards variability measures with evidence of prognostic power.

Drawing together insights from this study and consolidating guidance issued by others, we conclude that there are several areas in which methodological change in future prognostic studies of BP variability is needed if the field is to advance:

To improve the methodological quality of prognostic studies, all studies should attempt to follow-up all patients and carry out objective or independent outcome assessment. To facilitate the pooling of data, there is a need to standardise: (a) definitions of outcomes, particularly composite outcomes; (b) definitions of variability measures, particularly thresholds of night dipping, the dispersion measure which should be used, and what is meant by the term “morning surge”; and, (c) definitions of night and day. Standardisation would produce greater consistency across studies and this could enhance the opportunity for meta-analysis. To reduce confounding, relative risks should be adjusted for the appropriate mean BP (the mean must be defined across the same time period as the variability measure) and adjusted for treatment in studies of hypertensive patients. Clarity in reporting is also important, for example, by presenting patient characteristics, and by stating when variability was measured in studies of hypertensive patients i.e. whether under treatment or not. To reduce bias and avoid problems with categorisation, categorical expressions of relative risks would benefit from being accompanied by continuous analyses. Finally, to increase the evidence base of the prognostic value of BP variability, it would be helpful if individual patient data were made available and if research efforts probed further, for example, by stratifying relative risks by age group, and evaluating the risk of other cardiovascular outcomes beyond the primary outcomes of interest.

## Supporting Information

S1 PRISMA Checklist PRISMA checklist. (DOC)

S1 Appendix Search strategy (to 11 April 2013). (DOCX)

S2 Appendix List of extracted data. (DOCX)

S3 Appendix Study protocol. (DOC)

S1 Table List of full-text excluded articles. Only one reason is listed.
(DOCX)

S2 Table References of the studies included in the systematic review. References are grouped by study and are listed alphabetically.
(DOCX)

S3 Table Methodological limitations of the included studies. Evaluated by individual paper.
(DOCX)

S4 Table Numbers of studies reporting 24-hour blood pressure variability measures as a prognostic index of cardiovascular events: categorical expressions of relative risks. Includes studies reporting relative risks/hazard ratios based on pooled data from other studies.
(DOCX)

S5 Table Comparing predictive power of corresponding systolic and diastolic measures with relative risks scaled per 1 SD increase. Categorical expressions of relative risks. G—general; M—mixed; H—hypertensive; HD—hypertensive, diabetes; SD—standard deviation; ARV—average real variability; RR—relative risk. Relative risks:>1 increased risk;< 1 reduced risk.
(DOCX)
