# Supplementary material for: Heterogeneity of Prognostic Studies of 24-Hour Blood Pressure Variability: Systematic Review and Meta-Analysis
Source: PLoS One. 2015 May 18;10(5):e0126375. doi: 10.1371/journal.pone.0126375 (PMC4435972; doi:10.1371/journal.pone.0126375)
Supplement: S1 Appendix — (DOCX) [file pone.0126375.s002.docx]

**S1 Appendix. Search strategy (to 11 April 2013)**

| Search Question:  P = Cardiovascular disease  E = Blood pressure variability O = Prognosis / RCTs | | | |
| --- | --- | --- | --- |
| Databases searched:  Cochrane Library  Embase  Medline | Year range:  Issue 3, 2013  1980 – present  1946 – present | No records retrieved:  272  2591  1898 | Current update:  21  688  389 |
| Total no records retrieved:  4761 | No duplicates removed:  1737 | Final total:  3024 | Current update:  549 |
| Limits:  Human | | | |
| Medline search strategy:   \| \| 1 \| ((blood pressure or bp or sbp or dbp) adj5 (variation* or variab*)).tw. \| 8215 \| \| --- \| --- \| --- \| \| 2 \| ((morning or day* or diurnal or evening or night* or nocturnal) adj3 (variation* or variab*)).tw. \| 12979 \| \| 3 \| ((morning or day* or diurnal) adj3 surg*).tw. \| 25809 \| \| 4 \| ((evening or night* or nocturnal) adj3 (dip* or difference or decline or decrease*)).tw. \| 2827 \| \| 5 \| ("visit to visit" or "reading to reading").tw. \| 532 \| \| 6 \| ("night or day" or "day to night" or night?day or day?night).tw. \| 4965 \| \| 7 \| 1 or 2 or 3 or 4 or 5 or 6 \| 53102 \| \| 8 \| (blood pressure or bp or sbp or dbp).tw. \| 298536 \| \| 9 \| 7 and 8 \| 10362 \| \| 10 \| ((morning or day* or diurnal) adj (blood pressure or bp or sbp or dbp)).tw. \| 1600 \| \| 11 \| ((evening or night* or nocturnal) adj (blood pressure or bp or sbp or dbp)).tw. \| 1313 \| \| 12 \| 10 and 11 \| 414 \| \| 13 \| ((blood pressure or bp or sbp or dbp) adj3 (surg* or dip*)).tw. \| 1536 \| \| 14 \| (morning or evening or night*).tw. \| 85656 \| \| 15 \| 13 and 14 \| 548 \| \| 16 \| morning hypertension.tw. \| 77 \| \| 17 \| 9 or 12 or 15 or 16 \| 10621 \| \| 18 \| Cardiovascular Diseases/ \| 91283 \| \| 19 \| heart diseases/ or vascular diseases/ \| 78535 \| \| 20 \| exp Heart Failure/ \| 80716 \| \| 21 \| exp Myocardial Ischemia/ \| 339573 \| \| 22 \| Peripheral Vascular Diseases/ \| 10591 \| \| 23 \| exp Stroke/ \| 76081 \| \| 24 \| exp Brain Ischemia/ \| 76841 \| \| 25 \| ((cardiovascular or cardiac or heart or myocardial or vascular) adj3 disease*).tw. \| 249877 \| \| 26 \| ((heart or cardiac) adj failure).tw. \| 105355 \| \| 27 \| ((myocardial or cardiac or heart) adj (infarct* or ishemi* or ischaemi*)).tw. \| 140805 \| \| 28 \| (peripheral arterial disease* or peripheral vascular disease*).tw. \| 12420 \| \| 29 \| (stroke or cva or cerebrovascular accident* or tia or trans* isch* attack*).tw. \| 138758 \| \| 30 \| ((brain or cerebr*) adj (infarct* or ishemi* or ischaemi*)).tw. \| 16633 \| \| 31 \| 18 or 19 or 20 or 21 or 22 or 23 or 24 or 25 or 26 or 27 or 28 or 29 or 30 \| 906785 \| \| 32 \| 17 and 31 \| 2194 \| \| 33 \| Prognosis/ \| 335166 \| \| 34 \| prognos*.tw. \| 336129 \| \| 35 \| Mortality/ \| 32938 \| \| 36 \| mortality.fs. \| 386955 \| \| 37 \| (mortality or deaths).tw. \| 487356 \| \| 38 \| risk*.ti. \| 265381 \| \| 39 \| risk factor*.tw. \| 312577 \| \| 40 \| Risk Factors/ \| 515059 \| \| 41 \| proportional hazards models/ \| 38313 \| \| 42 \| (hazard ratio* or hazard model*).tw. \| 41167 \| \| 43 \| exp Cohort Studies/ \| 1245408 \| \| 44 \| ((cohort or follow up or longitudinal) adj stud*).tw. \| 145574 \| \| 45 \| 33 or 34 or 35 or 36 or 37 or 38 or 39 or 40 or 41 or 42 or 43 or 44 \| 2596944 \| \| 46 \| 32 and 45 \| 1373 \| \| 47 \| cardiovascular risk*.tw. \| 37448 \| \| 48 \| 17 and 47 \| 591 \| \| 49 \| randomized controlled trial.pt. \| 346677 \| \| 50 \| controlled clinical trial.pt. \| 85722 \| \| 51 \| randomized.ab. \| 264499 \| \| 52 \| placebo.ab. \| 143200 \| \| 53 \| drug therapy.fs. \| 1600088 \| \| 54 \| randomly.ab. \| 192601 \| \| 55 \| trial.ab. \| 273063 \| \| 56 \| groups.ab. \| 1242772 \| \| 57 \| 49 or 50 or 51 or 52 or 53 or 54 or 55 or 56 \| 3098705 \| \| 58 \| 32 and 57 \| 882 \| \| 59 \| 46 or 48 or 58 \| 1963 \| \| 60 \| exp animals/ not humans/ \| 3800177 \| \| 61 \| 59 not 60 \| 1898 \| \| 62 \| (2011* or 2012* or 2013*).dp,ed,yr. \| 2670349 \| \| 63 \| 61 and 62 \| 389 \| \| \| --- \| --- \| --- \| --- \| --- \| --- \| --- \| --- \| --- \| --- \| --- \| --- \| --- \| --- \| --- \| --- \| --- \| --- \| --- \| --- \| --- \| --- \| --- \| --- \| --- \| --- \| --- \| --- \| --- \| --- \| --- \| --- \| --- \| --- \| --- \| --- \| --- \| --- \| --- \| --- \| --- \| --- \| --- \| --- \| --- \| --- \| --- \| --- \| --- \| --- \| --- \| --- \| --- \| --- \| --- \| --- \| --- \| --- \| --- \| --- \| --- \| --- \| --- \| --- \| --- \| --- \| --- \| --- \| --- \| --- \| --- \| --- \| --- \| --- \| --- \| --- \| --- \| --- \| --- \| --- \| --- \| --- \| --- \| --- \| --- \| --- \| --- \| --- \| --- \| --- \| --- \| --- \| --- \| --- \| --- \| --- \| --- \| --- \| --- \| --- \| --- \| --- \| --- \| --- \| --- \| --- \| --- \| --- \| --- \| --- \| --- \| --- \| --- \| --- \| --- \| --- \| --- \| --- \| --- \| --- \| --- \| --- \| --- \| --- \| --- \| --- \| --- \| --- \| --- \| --- \| --- \| --- \| --- \| --- \| --- \| --- \| --- \| --- \| --- \| --- \| --- \| --- \| --- \| --- \| --- \| --- \| --- \| --- \| --- \| --- \| --- \| --- \| --- \| --- \| --- \| --- \| --- \| --- \| --- \| --- \| --- \| --- \| --- \| --- \| --- \| --- \| --- \| --- \| --- \| --- \| --- \| --- \| --- \| --- \| --- \| --- \| --- \| --- \| --- \| --- \| --- \| --- \| --- \| --- \| --- \| --- \| --- \| --- \| --- \| --- \| | | | |
