# Supplementary material for: Heterogeneity of Prognostic Studies of 24-Hour Blood Pressure Variability: Systematic Review and Meta-Analysis
Source: PLoS One. 2015 May 18;10(5):e0126375. doi: 10.1371/journal.pone.0126375 (PMC4435972; doi:10.1371/journal.pone.0126375)
Supplement: S2 Appendix — (DOCX) [file pone.0126375.s003.docx]

**S2 Appendix. List of extracted data**

Type of study

Source of patient population

Type of patient population

Number of patients

24-hour BP variability measure

Timing of measurement

Definition of day-time and night-time

BP measured (systolic, diastolic or both together)

Frequency of BP data measured (high for ambulatory or low for within-visit)

Outcome

Qualifying medical conditions for outcome

Relative risk with confidence intervals (Data given as beta coefficient and SE were converted to relative risks and confidence interval)

Expression of relative risk (as a continuous measure or categorical measure)

Scaling factor for relative risks expressed as continuous variables

Definitions of categories for relative risks expressed as categorical variables

Factors adjusted in calculation of relative risks
