# Supplementary material for: Heterogeneity of Prognostic Studies of 24-Hour Blood Pressure Variability: Systematic Review and Meta-Analysis
Source: PLoS One. 2015 May 18;10(5):e0126375. doi: 10.1371/journal.pone.0126375 (PMC4435972; doi:10.1371/journal.pone.0126375)
Supplement: S3 Appendix — (DOC) [file pone.0126375.s004.doc]

**S3 Appendix. Study protocol**

**LATEST UPDATE**

December 2013

**TITLE**

A systematic review of the prognostic value of the relationship between 24-hour blood pressure variability and cardiovascular outcome

**QUESTIONS**

How is 24-hour blood pressure (BP) variability defined?

What is the prognostic value of 24-hour BP variability with regards to cardiovascular events?

**OBJECTIVES**

1. To explore the variation of methods of analysis of the prognostic value of 24-hour BP variability

2. To summarise the best evidence on the prognostic value of 24-hour BP variability in predicting cardiovascular morbidity and/ or mortality

3. To determine the prognostic value of 24-hour BP variability in predicting sub types of CVD including stroke and acute coronary events

**METHODS**

Studies included in the review must have the following characteristics:

**Inclusion criteria – POPULATION**

Adults (>18), male or female

Hypertensive or normotensive, treated or untreated

With known or unknown cardiovascular co-morbidities

**Inclusion criteria - INDICATOR**

Systolic or diastolic BP variability or both

Ambulatory BP measurement, during the day, night or both

Any measure of 24-hour BP variability

Follow-up of at least one year

**Inclusion criteria - STUDY DESIGNS**

Randomised controlled trials

Observational cohort studies

**Exclusion criteria - STUDY DESIGNS**

Retrospective studies

Case control trials

**Inclusion criteria – OUTCOME MEASUREMENTS**

**Cardiovascular events**

**a. Coronary artery disease encompasses**

- Acute coronary syndrome: which includes – STEMI, NSTEMI and unstable angina. Myocardial infarction (diagnosed with 2/3 of a. typical chest pain b. ECG changes c. biochemical markers of ischaemia)
- An objective diagnosis of angina pectoris e.g. angiographic evidence of significant obstruction, revascularisation procedures
- An objective diagnosis of congestive heart failure

**b. Cerebrovascular disease**

- Focal neurological deficit lasting > 24hrs (to include retinovascular events)
- TIA i.e. focal neurological deficit lasting < 24hrs

Methodological quality will be assessed using Heneghan and Badenock (2006).

**PROTOCOL HISTORY**

**February 2010**

Date of original protocol.

**Amendments of February 2011**

Study objectives and research questions

The protocol was originally drawn up, prior to the first literature search (23 February 2010), reflecting our intention to carry out a systematic review and meta-analysis of the prognostic value of a few measures of BP variability within 24 hours. However, examining the data from this first search (meeting report, August 2010), we discovered a diversity of methods and multiple measures which would limit our ability to pool data and draw conclusions. We considered these preliminary findings had important implications, so we amended the protocol to reflect a broader focus of the research to also explore the variation of methods.

We would attempt to address the pre-specified objectives 1 and 2, within the constraints of the data available. Variability indexes (in Inclusion criteria for inclusion and exclusion of studies) would be ANY short-term measure of BP variability. We referred to “short-term blood pressure variability” to encompass Ambulatory BP, home self-monitoring of BP or repetitive office measurements (3+).

Quality assessment

We also had concerns about the quality assessment criteria of Altman [1], being open to interpretation (see email from AW, 7 Aug 2010). We contacted Douglas Altman requesting further clarification and he replied (email 13 Sept 2010) saying that his 2001 paper was not intended to present a quality scoring system. We decided to apply the quality assessment criteria of Heneghan and Badenock [2], which encapsulates the issues described in the Altman paper. The Heneghan and Badenock criteria [2] had been applied recently [3].

**Amendments of December 2013**

Blood pressure measurement

We were advised that, as it was inappropriate to study together different short-term BP measurements, we should focus on ambulatory BP measurement. Although we would not have pooled data of different short-term BP measures, we accepted that analysing the methods of all short-term studies together would be confusing. Given the variation in terminology concerning measurement of BP variability based on ambulatory measurement, we decided to refer to “24-hour” variability.

**REFERENCES**

1. Altman DG. Systematic reviews of evaluations of prognostic variables. BMJ 2001; 323: 224-228.
2. Heneghan C, Badenock D Evidence-based medicine toolkit, 2nd ed. 2006 Oxford Blackwell Publishing
3. Ward AM, Takahashi O, Stevens R, Heneghan C J. Home measurement of blood pressure and cardiovascular disease: systematic review and meta-analysis of prospective studies. J Hypertens. 2012; 30:449-56
