# Supplementary material for: Heterogeneity of Prognostic Studies of 24-Hour Blood Pressure Variability: Systematic Review and Meta-Analysis
Source: PLoS One. 2015 May 18;10(5):e0126375. doi: 10.1371/journal.pone.0126375 (PMC4435972; doi:10.1371/journal.pone.0126375)
Supplement: S1 Table — Only one reason is listed. (DOCX) [file pone.0126375.s005.docx]

**S1 Table. List of full-text excluded articles**

| **No** | **First author** | **Year** | **Reason for exclusion from review*** |
| --- | --- | --- | --- |
| 1 | Ali | 2011 | Not a prognostic study |
| 2 | Berry | 2011 | Not an outcome of interest |
| 3 | Bjorklund | 2004 | Duplicate/overlapping population |
| 4 | Brunelli | 2008 | Not 24 hour BPV |
| 5 | Cornelissen | 2002 | Duplicate/overlapping population |
| 6 | Di Iorio | 1999 | Not a prognostic study |
| 7 | Dolan | 2005 | Not 24-hour BPV |
| 8 | Dolan | 2010 | Editorial |
| 9 | Eguchi | 2008 | Duplicate/overlapping population |
| 10 | Geeganage | 2011 | Insufficient follow-up |
| 11 | Grove | 1997 | Not 24-hour BPV |
| 12 | Hermida | 2010 | Not 24-hour BPV |
| 13 | Hermida | 2013 | Letter |
| 14 | Hoshide | 2002 | Duplicate/overlapping population |
| 15 | Kario | 2003 | Duplicate/overlapping population |
| 16 | Kario | 2004 | Editorial |
| 17 | Kario | 2011 | Duplicate/overlapping population |
| 18 | Kikuya | 2000 | Duplicate/overlapping population |
| 19 | Kisaka | 2012 | Not an prognostic study |
| 20 | Ko | 2010 | Not an outcome of interest |
| 21 | Kobrin | 1984 | Did not measure BPV |
| 22 | Kukla | 1998 | Not a prognostic study |
| 23 | Li | 2008 | Duplicate/overlapping population |
| 24 | Manios | 2011 | Cross-sectional study |
| 25 | Nakamura | 1995 | Not a prognostic study |
| 26 | O'Brien | 1988 | Letter |
| 27 | Ohkubo | 1997 | Duplicate/overlapping population |
| 28 | Otsuka | 1996 | Duplicate/overlapping population |
| 29 | Palatini | 1992 | Not an outcome of interest |
| 30 | Parati | 1987 | Not an outcome of interest |
| 31 | Pickering | 2007 | Duplicate/overlapping population |
| 32 | Sega | 2002 | Not an outcome of interest |
| 33 | Shimizu | 2011 | Duplicate/overlapping population |
| 34 | Shin | 2007 | Not an outcome of interest |
| 35 | Staessen | 1999 | Duplicate/overlapping population |
| 36 | Tozawa | 1999 | Not 24-hour BPV |
| 37 | Vaz-de-Melo | 2010 | Not a prognostic study |
| 38 | Verdecchia | 1996 | Duplicate/overlapping population |
| 39 | Webb | 2010 | Not a prognostic study |
| 40 | Zis | 2012 | Letter |
| 41 | Zis | 2013 | Not an outcome of interest |

Ali K, Leong KM, Houlder S, Getov S, Lee R, Rajkumar C. The relationship between dipping profile in blood pressure and neurologic deficit in early acute ischemic stroke.J Stroke Cerebrovasc Dis. 2011 Jan-Feb;20(1):10-5. doi: 10.1016/j.jstrokecerebrovasdis.2009.09.012. Epub 2010 Jun 9.

Berry M, Fourcade J, et al. European Heart Journal Conference: European Society of Cardiology, ESC Congress 2011 Paris France. Conference Start: 20110827 Conference End: 20110831. Conference Publication: (var.pagings). 32: 135.

Björklund K, Lind L, Zethelius B, Berglund L, Lithell H. Prognostic significance of 24-h ambulatory blood pressure characteristics for cardiovascular morbidity in a population of elderly men. J Hypertens. 2004 Sep;22(9):1691-7.

Brunelli SM, Thadhani RI, Lynch KE, Ankers ED, Joffe MM, Boston R, Chang Y, Feldman HI. Association between long-term blood pressure variability and mortality among incident hemodialysis patients. Am J Kidney Dis. 2008 Oct;52(4):716-26. doi: 10.1053/j.ajkd.2008.04.032. Epub 2008 Aug 27.

Cornelissen G, Otsuka K, Bakken EE, Halberg F, Siegelova J, Fiser B. CHAT (circadian hyper-amplitude-tension) and CSDD-HR (Circadian standard deviation deficit of heart rate): separate, synergistic vascular disease risks? SCRIPTA MEDICA (BRNO) – 75 (2): 87–94, *April 2002*

Di Iorio A, Marini E, Lupinetti M, Zito M, Abate G. Blood pressure rhythm and prevalence of vascular events in hypertensive subjects. Age Ageing. 1999 Jan;28(1):23-8.

Dolan E, Stanton A, Thijs L, Hinedi K, Atkins N, McClory S, Den Hond E, McCormack P, Staessen JA, O'Brien E. Superiority of ambulatory over clinic blood pressure measurement in predicting mortality: the Dublin outcome study.Hypertension. 2005 Jul;46(1):156-61. Epub 2005 Jun 6.

Dolan E, O'Brien E. Blood pressure variability: clarity for clinical practice. Hypertension. 2010 Aug;56(2):179-81. doi: 10.1161/HYPERTENSIONAHA.110.154708. Epub 2010 Jul 6.

Eguchi K, Pickering TG, Hoshide S, Ishikawa J, Ishikawa S, Schwartz JE, Shimada K, Kario K. Ambulatory blood pressure is a better marker than clinic blood pressure in predicting cardiovascular events in patients with/without type 2 diabetes. Am J Hypertens. 2008 Apr;21(4):443-50. doi: 10.1038/ajh.2008.4. Epub 2008 Feb 21.

Geeganage C, Tracy M, England T, Sare G, Moulin T, Woimant F, Christensen H, De Deyn PP, Leys D, O'Neill D, Ringelstein EB, Bath PM; for TAIST Investigators. Relationship between baseline blood pressure parameters (including mean pressure, pulse pressure, and variability) and early outcome after stroke: data from the Tinzaparin in Acute Ischaemic Stroke Trial (TAIST). Stroke. 2011 Feb;42(2):491-3. doi: 10.1161/STROKEAHA.110.596163. Epub 2010 Dec 23.

Grove JS, Reed DM, Yano K, Hwang LJ. Variability in systolic blood pressure--a risk factor for coronary heart disease? Am J Epidemiol. 1997 May 1;145(9):771-6.

Hermida RC, Ayala DE, Mojón A, Fernández JR. Influence of circadian time of hypertension treatment on cardiovascular risk: results of the MAPEC study. Chronobiol Int. 2010 Sep;27(8):1629-51. doi: 10.3109/07420528.2010.510230.

Hermida RC, Ayala DE, Mojón A, Fernández JR. Blunted sleep-time relative blood pressure decline increases cardiovascular risk independent of blood pressure level--the "normotensive non-dipper" paradox. Chronobiol Int. 2013 Mar;30(1-2):87-98. doi: 10.3109/07420528.2012.701127. Epub 2012 Oct 5.

Hoshide Y, Kario K, Schwartz JE, Hoshide S, Pickering TG, Shimada K. Incomplete benefit of antihypertensive therapy on stroke reduction in older hypertensives with abnormal nocturnal blood pressure dipping (extreme-dippers and reverse-dippers). Am J Hypertens. 2002 Oct;15(10 Pt 1):844-50.

Kario K, Shimada K, Pickering TG. Clinical implication of morning blood pressure surge in hypertension. J Cardiovasc Pharmacol. 2003 Dec;42 Suppl 1:S87-91.

Kario K. Blood pressure variability in hypertension: a possible cardiovascular risk factor. Am J Hypertens. 2004 Nov;17(11 Pt 1):1075-6.

Kikuya M, Hozawa A, Ohokubo T, Tsuji I, Michimata M, Matsubara M, Ota M, Nagai K, Araki T, Satoh H, Ito S, Hisamichi S, Imai Y. Prognostic significance of blood pressure and heart rate variabilities: the Ohasama study. Hypertension. 2000 Nov;36(5):901-6.

Ko Y, Park JH, Yang MH, Ko S-B, Han M-K, Oh CW, Lee JS. Juneyoung Lee, PhD; Hee-Joon Bae,The significance of blood pressure variability for the development of hemorrhagic transformation in acute ischemic stroke. Stroke . 2010;41:2512-2518.

Kobrin I, Oigman W, Kumar A, Ventura HO, Messerli FH, Frohlich ED, Dunn FG. Diurnal variation of blood pressure in elderly patients with essential hypertension. J Am Geriatr Soc. 1984 Dec;32(12):896-9.

Kukla C, Sander D, Schwarze J, Wittich I, Klingelhöfer J. Changes of circadian blood pressure patterns are associated with the occurence of lucunar infarction. Arch Neurol. 1998 May;55(5):683-8.

Li Y, Boggia J, Thijs L, Hansen TW, Kikuya M, Björklund-Bodegård K, Richart T, Ohkubo T, Kuznetsova T, Torp-Pedersen C, Lind L, Ibsen H, Imai Y, Wang J, Sandoya E, O'brien E, Staessen JA; International Database on Ambulatory Blood Pressure Monitoring in relation to Cardiovascular Outcomes Investigators. Is blood pressure during the night more predictive of cardiovascular outcome than during the day? Blood Press Monit. 2008 Jun;13(3):145-7. doi: 10.1097/MBP.0b013e3282fd16cc.

Manios E, Stamatelopoulos K, Tsivgoulis G, Barlas G, Koroboki E, Tsagalis G, Michas F, Vemmos K, Zakopoulos N. Time rate of blood pressure variation: a new factor associated with coronary atherosclerosis. J Hypertens. 2011 Jun;29(6):1109-14. doi: 10.1097/HJH.0b013e3283454ff4.

Nakamura K, Oita J, Yamaguchi T. Nocturnal blood pressure dip in stroke survivors. A pilot study. Stroke. 1995 Aug;26(8):1373-8.

O'Brien E, Sheridan J, O'Malley K. Dippers and non-dippers. Lancet. 1988 Aug 13;2(8607):397.

Ohkubo T, Imai Y, Tsuji I, Nagai K, Watanabe N, Minami N, Kato J, Kikuchi N, Nishiyama A, Aihara A, Sekino M, Satoh H, Hisamichi S. Relation between nocturnal decline in blood pressure and mortality. The Ohasama Study. Am J Hypertens. 1997 Nov;10(11):1201-7.

Otsuka K, Cornélissen G, Halberg F, Oehlerts G. Excessive circadian amplitude of blood pressure increases risk of ischaemic stroke and nephropathy. J Med Eng Technol. 1997 Jan-Feb;21(1):23-30.

Palatini P, Penzo M, Racioppa A, Zugno E, Guzzardi G, Anaclerio M, Pessina AC. Clinical relevance of nighttime blood pressure and of daytime blood pressure variability. Arch Intern Med. 1992 Sep;152(9):1855-60.

Pickering T, Schwartz J, Verdecchia P, Imai Y, Kario K, Eguchi K, Pierdomenico S, Ohkubo T, Wing L. Prediction of strokes versus cardiac events by ambulatory monitoring of blood pressure: results from an international database. Blood Press Monit. 2007 Dec;12(6):397-9. doi: 10.1097/MBP.0b013e3282411a12.

Sega R, Corrao G, Bombelli M, Beltrame L, Facchetti R, Grassi G, Ferrario M, Mancia G. Blood pressure variability and organ damage in a general population: results from the PAMELA study (Pressioni Arteriose Monitorate E Loro Associazioni). Hypertension. 2002 Feb;39(2 Pt 2):710-4.

Shimizu M, Ishikawa J, Yano Y, Hoshide S, Shimada K, Kario K.The relationship between the morning blood pressure surge and low-grade inflammation on silent cerebral infarct and clinical stroke events. Atherosclerosis. 2011 Nov;219(1):316-21. doi: 10.1016/j.atherosclerosis.2011.06.030. Epub 2011 Jun 23.

Shin J, Kline S, Moore M, Gong Y, Bhanderi V, Schmalfuss CM, Johnson JA, Schofield RS. Association of diurnal blood pressure pattern with risk of hospitalization or death in men with heart failure. J Card Fail. 2007 Oct;13(8):656-62.

Staessen JA, Thijs L, Fagard R, O'Brien ET, Clement D, de Leeuw PW, Mancia G, Nachev C, Palatini P, Parati G, Tuomilehto J, Webster J. Predicting cardiovascular risk using conventional vs ambulatory blood pressure in older patients with systolic hypertension. Systolic Hypertension in Europe Trial Investigators. JAMA. 1999 Aug 11;282(6):539-46.

Tozawa M, Iseki K, Yoshi S, Fukiyama K.Blood pressure variability as an adverse prognostic risk factor in end-stage renal disease. Nephrol Dial Transplant. 1999 Aug;14(8):1976-81.

Vaz-de-Melo RO, Toledo JC, Loureiro AA, Cipullo JP, Moreno Júnior H, Martin JF. [Absence of nocturnal dipping is associated with stroke and myocardium infarction].[Article in Portuguese] Arq Bras Cardiol. 2010 Jan;94(1):79-85

Verdecchia P, Borgioni C, Ciucci A, Gattobigio R, Schillaci G, Sacchi N, Santucci A, Santucci C, Reboldi G, Porcellati C. Prognostic significance of blood pressure variability in essential hypertension. Blood Press Monit. 1996 Feb;1(1):3-11.

Webb AJ, Fischer U, Mehta Z, Rothwell PM. Effects of antihypertensive-drug class on interindividual variation in blood pressure and risk of stroke: a systematic review and meta-analysis. Lancet. 2010 Mar 13;375(9718):906-15. doi: 10.1016/S0140-6736(10)60235-8.

Zis P, Spengos K, Manios E, Vemmos K, Zis V, Dimopoulos MA, Zakopoulos N. Ambulatory blood pressure monitoring in acute stroke: the importance of time rate of blood pressure variation. Blood Press Monit. 2012 Oct;17(5):220-1; author reply 221-2. doi: 10.1097/MBP.0b013e3283588e8a.

Zis P, Vemmos K, Spengos K, Manios E, Zis V, Dimopoulos MA, Zakopoulos N. Ambulatory blood pressure monitoring in acute stroke: pathophysiology of the time rate of blood pressure variation and association with the 1-year outcome. Blood Press Monit. 2013 Apr;18(2):94-100. doi: 10.1097/MBP.0b013e32835ebc3f.
