# Supplementary material for: Heterogeneity of Prognostic Studies of 24-Hour Blood Pressure Variability: Systematic Review and Meta-Analysis
Source: PLoS One. 2015 May 18;10(5):e0126375. doi: 10.1371/journal.pone.0126375 (PMC4435972; doi:10.1371/journal.pone.0126375)
Supplement: S2 Table — References are grouped by study and are listed alphabetically. (DOCX) [file pone.0126375.s006.docx]

**S2 Table. References of the studies included in the systematic review**

| **Ref** | **Paper** |
| --- | --- |
| W1 | **Chieti University** |
| W1a | Pierdomenico SD, Lapenna D, Bucci A, Manente BM, Mancini M, Cuccurullo F et al. Blood pressure variability and prognosis in uncomplicated mild hypertension. Am Heart J 2005; 149:934-938. |
| W1b | Pierdomenico SD, Lapenna D, Di Tommaso R, Di Carlo S, Esposito AL, Di Mascio R et al.Blood pressure variability and cardiovascular risk in treated hypertensive patients. AJH 2006; 19:991-997 |
| W1c | Pierdomenico SD, Di Nicola M, Esposito AL, Di Mascio R, Ballone E, Lapenna D et al. Prognostic value of different indices of blood pressure variability in hypertensive patients. Am J Hypertens 2009; 22: 842-847. |
| W2 | **International Database on Ambulatory Blood Pressure Monitoring in Relation to Cardiovascular Outcomes (IDACO)** |
| W2a | Boggia J, Li Y, Thijs L, Hansen TW, Kikuya M, Björklund-Bodegård K, Richart T et al; International Database on Ambulatory blood pressure monitoring in relation to Cardiovascular Outcomes (IDACO) investigators. Prognostic accuracy of day versus night ambulatory blood pressure: a cohort study. Lancet 2007; 370: 1219-1229. |
| W2b | Fagard RH, Celis H, Thijs L, Staessen JA, Clement DL, De Buyzere ML et al. Daytime and nighttime blood pressure as predictors of death and cause-specific cardiovascular events in hypertension. Hypertension 2008; 51: 55-61. |
| W2c | Fagard RH, Thijs L, Staessen JA, Clement DL, De Buyzere ML, De Bacquer DA. Night-day blood pressure ratio and dipping pattern as predictors of death and cardiovascular events in hypertension. J Hum Hypertens 2009; 23: 645-653. |
| W2d | Hansen TW, Jeppensen J, Rasmussen S, Ibsen H, Torp_Pedersen C. Ambulatory blood pressure monitoring and risk of cardiovascular disease: A population based study. AJH 2006; 19: 243-250. |
| W2e | Hansen TW, Thijs L, Li Y, Boggia J, Kikuya M, Björkland-Bodegård K et al. Prognostic value of reading blood pressure variability over 24 hours in 8938 subjects from 11 populations. Hypertension 2010; 55: 1049-1057. |
| W2f | Li Y, Thijs L, Hansen TW. Prognostic value of the morning surge in 5645 subjects from 8 populations. Hypertension 2010; 55: 1040-1048. |
| W2g | Metoki H, Ohkubo T, Kikuya M, Asayama K, Obara T, Hashimoto J et al. Prognostic significance for stroke of a morning pressor surge and a nocturnal blood pressure decline: the Ohasama study. Hypertension 2006; 47: 149-154. |
| W2h | Ohkubo T, Imai Y, Tsuji I, Nagai K, Watanabe N, Minami N et al. Relation between nocturnal decline in blood pressure and mortality. The Ohasama Study. Am J Hypertens. 1997 10:1201-7. |
| W2i | Ohkubo T, Hozawa A,Yamaguchi J, Kikuya M, Ohmori K, Michimata M et al. Prognostic significance of the nocturnal decline in blood pressure in individuals with and without high 24-h blood pressure: the Ohasama study. J Hypertens 2002; 20: 2183-2189. |
| W2j | Pringle E, Phillips C, Thijs L, Davidson C, Staessen JA, de Leeue PW et al.Systolic blood pressure variability as a risk factor for stroke and cardiovascular mortality in the elderly hypertensive population. J Hypertens 2003; 21: 2251-2257. |
| W3 | **Jichi Medical School** |
| W3a | Eguchi K, Ishikawa S, Pickering T, Schwartz JE, Shimada K, Kario K. Night time blood pressure variability is a strong predictor for cardiovascular events in patients with type 2 diabetes. Am J Hypertens 2009; 22: 46-51. |
| W3b | Kabutoya T, Hoshide S, Ishikawa J, Eguchi K, Shimada K, Kario K.The effect of pulse rate and blood pressure dipping status on the risk of stroke and cardiovascular disease in Japanese hypertensive patients. Am J Hypertens 2010; 23: 749-755. |
| W3c | Kario K, Pickering TG, Matsuo T, Hoshide S, Schwartz JE, Shimada K. Stroke prognosis and abnormal nocturnal blood pressure falls in older hypertensives. Hypertension 2001; 38: 852-857. |
| W3d | Kario K, Pickering TG, Umeda Y. Morning surge in blood pressure as a predictor of silent and clinical cerebrovascular disease in elderly hypertensives: a prospective study. Circulation 2003; 107: 1401-1406. |
| W4 | **Progetto Ipertensione Umbria Monitoraggio Ambulatoriale (PIUMA)** |
| W4a | Verdecchia P, Porcellati C, Schillaci G, Borgioni C, Ciucci A, Battistelli M et al. Ambulatory blood pressure. An independent predictor of prognosis in essential hypertension. Hypertension 1994; 24:793-801. |
| W4b | Verdecchia P, Schillaci G, Borgioni C Ciucci A, Gattobigio R, Guerrieri M et al. Altered circadian blood pressure profile and prognosis. Blood Press Monit 1997; 2: 347-352. |
| W4c | Verdecchia P, Angeli F, Gattoibio R, Rapicetta C, Reboldi G. Impact of blood pressure variability on cardiac and cerebrovascular complications in hypertension. AJH 2007; 20: 154-161. |
| W4d | Verdecchia P, Angeli F, Borgioni C, Repaci S, Guerrieri M, Andreani F et al. Prognostic value of circadian blood pressure changes in relation to differing measures of day and night. J Am Soc Hypertens 2008; 2:88-96 |
| W4e | Verdecchia P, Angeli F, Mazzotta G, Garofoli M, Ramundo E, Gentile G, Ambrosio G, Reboldi G.Day-night dip and early-morning surge in blood pressure in hypertension: prognostic implications. Hypertension. 2012 Jul;60:34-42. |
| W5 | **Hadassah Hebrew University** |
| W5a | Ben-Dov IZ, Kark JD, Ben-Ishay D, Mekler J, Ben-Arie L, Bursztyn M. Predictors of all-cause mortality in clinical ambulatory monitoring: unique aspects of blood pressure during sleep. Hypertension. 2007 Jun;49(6):1235-41. Epub 2007 Mar 26. |
| W4b | Israel S, Israel A, Ben-Dov IZ, Bursztyn M. The morning blood pressure surge and all-cause mortality in patients referred for ambulatory blood pressure monitoring. Am J Hypertens. 2011 Jul;24:796-801. |
|  | **Other studies** |
| W6 | Amici A, Cicconetti P, Sagrafoli C, Baratta A, Passador P, Pecci T et al. Exaggerated morning blood pressure surge and cardiovascular events. A 5-year longitudinal study in normotensive and well-controlled hypretensive elderly. Arch Gerontol Geriatr 2009; 49: e105-e109. |
| W7 | Astrup, Nielsen FS, Rossing P, Ali S, Kastrup J, Smidt UM et al. Predictors of mortality in patients with type 2 diabetes with or without diabetic nephropathy: a follow-up study. J Hypertens 2007; 25: 2479-2485 |
| W8 | Bouhanick B, Bongard V, Amar J, Bousquel S, Chamontin B. Prognostic value of nocturnal blood pressure and reverse-dipping status on the occurrence of cardiovascular events in hypertensive diabetic patients. Diabetes Metab 2008; 34:560-567. |
| W9 | Brotman DJ, Davidson MB, Boumitri M, Vidt DG. Impaired diurnal blood pressure variation and all-cause mortality. Am J Hypertens. 2008; 21:92-7. |
| W10 | de la Sierra A, Banegas JR, Segura J, Gorostidi M, Ruilope LM; CARDIORISC Event Investigators. Ambulatory blood pressure monitoring and development of cardiovascular events in high-risk patients included in the Spanish ABPM registry: the CARDIORISC Event study. J Hypertens. 2012 Apr;30:713-9. |
| W11 | Eto M, Toba K, Akishita M, Kozaki K, Watanabe T, Kim S, Hashimoto M et al. Impact of blood pressure variability on cardiovascular events in elderly patients with hypertension. Hypertens Res 2005; 28:1-7. |
| W12 | Gosse P, Lasserre R, Minifie C, Lemetayer P, Clementy J, Blood pressure surge on rising. J Hypertens 2004; 22: 1113-1118. |
| W13 | Iqbal P, Stevenson L. Cardiovascular Outcomes in Patients with Normal and Abnormal 24-Hour Ambulatory Blood Pressure Monitoring. Int J Hypertens. 2011; 2011: 786912.Published online 2010 December 5. doi: 10.4061/2011/786912PMCID: PMC2997497 |
| W14 | Liu M, Takahashi H, Morita Y et al. Non dipping is a potential predictor of cardiovascular mortality and is associated with autonomic dysfunction of haemodialysis patients Nephrol Dial Transplant 2003; 18:563-569 |
| W15 | Mancia G, Bombelli M, Facchettu R, Madotto F, Corrao G, Trevano FQ et al. Long-term prognostic value of blood pressure variability in the general population: Results of the Pressioni Arteriose Monitorate e Loro Associazioni Study. Hypertension 2007; 49: 1265-1270. |
| W16 | Mena L, Pintos S, Queipo NV, Aizpúrua JA, Maestre G, Sulbarán T. A reliable index for the prognostic significance of blood pressure variability. J Hypertens. 2005 Mar;23(3):505-11. |
| W17 | Minutolo R, Agarwal R, Borrelli S, Chiodini P, Bellizzi V, Nappi F, Cianciaruso B, Zamboli P, Conte G, Gabbai FB, de Nicola L. Prognostic role of ambulatory blood pressure measurement in patients with chronic kidney disease. Arch Intern Med. 2011;171:1090-1098 |
| W18 | Muxfeldt ES, Lopes Cardoso CR, Salles GF. Prognostic value of nocturnal blood pressure reduction in resistant hypertension. Ach Intern Med 2009; 169: 874-880. |
| W19 | Nakamura K, Oita J, Yamaguchi T. Nocturnal blood pressure dip in stroke survivors: a pilot study. Stroke 1995; 26:1373-1378 |
| W20 | Nakano S, Tomohiko I, Furuya K, Madotto F, Corrao G, Trevano FQ et al. Ambulatory blood pressure level rather than dipper/nondipper status predicts vascular events in type 2 diabetic subjects. Hypertens Res 2004; 27: 647-656. |
| W21 | Otsuka K, Cornélissen G, Halberg F. Predictive value of blood pressure dipping and swinging with regard to vascular disease risk. Clin Drug Investig 1996; 11: 20-31. |
| W22 | Rothwell PM, Howard SC, Dolan E, O’Brien E, Dobson JE, Dahlöf et al. Prognostic significance of visit-to-visit variability, maximum systolic blood pressure and episodic hypertension. Lancet 2010;375: 895-905. |
| W23 | Sturrock ND, George E, Pound N, Stevenson J, Peck GM, Sowter H. Non-dipping circadian blood pressure and renal impairment are associated with increased mortality in diabetes mellitus. Diabet Med. 2000 May;17:360-4. |
| W24 | Zweiker R, Eber B, Schumacher M, Toplak H, Klein W. Non dipping related to CV events in essential hypertensive patients. Acta Med Austriaca 1994; 21: 86-89. |
