# Supplementary material for: Heterogeneity of Prognostic Studies of 24-Hour Blood Pressure Variability: Systematic Review and Meta-Analysis
Source: PLoS One. 2015 May 18;10(5):e0126375. doi: 10.1371/journal.pone.0126375 (PMC4435972; doi:10.1371/journal.pone.0126375)
Supplement: S3 Table — Evaluated by individual paper. (DOCX) [file pone.0126375.s007.docx]

**S3 Table. Methodological limitations of the included studies**

| **Quality criterion** | **References** | **Number of papers** |
| --- | --- | --- |
| Did not recruit subjects in the same setting | W2a, W2b, W2c, W2e, W2f, W2j,W3a, W3c, W3d, W5b, W6, W10 | 12 |
| Did not present clinical and demographic characteristics | W21, W24 | 2 |
| Did not adjust relative risks for appropriate mean blood pressure | W1a, W2i, W4d, W7, W8, W13, W14, W19, W21, W23, W24 | 11 |
| Follow-up less than 5 years | W1a, W1b, W1c, W2j, W3a, W3b, W3c, W3d, W10, W11, W14, W16, W17, W18, W19, W23, W24 | 17 |
| Did not report complete follow-up | W4e, W5a, W9, W10, W16, W17, W23 | 7 |
| Did not report objective or independent assessment of outcomes | W1b, W1c, W2a, W2d, W2e, W2f, W2g, W2h, W2i, W3a, W3c, W3d, W4a, W4b, W4c, W4d, W6, W7, W8, W11, W12, W13, W14, W15, W16, W17, W19, W20, W21, W24 | 30 |
