# Supplementary material for: Heterogeneity of Prognostic Studies of 24-Hour Blood Pressure Variability: Systematic Review and Meta-Analysis
Source: PLoS One. 2015 May 18;10(5):e0126375. doi: 10.1371/journal.pone.0126375 (PMC4435972; doi:10.1371/journal.pone.0126375)
Supplement: S4 Table — Includes studies reporting relative risks/hazard ratios based on pooled data from other studies. (DOCX) [file pone.0126375.s008.docx]

**S4 Table. Numbers of studies reporting 24-hour blood pressure variability measures as a prognostic index of cardiovascular events: categorical expressions of relative risks**

| **Class** | **BP** | **Timing** | **Expression** | **All-cause mortality** | **CV mortality** | **All CV events** | **Stroke** | **CHD** |
| --- | --- | --- | --- | --- | --- | --- | --- | --- |
| **Hypertensive populations** | | | | | | | | |
| SD | Systolic | Day | Cat(HvL) | 0 | 0 | 1 | 0 | 0 |
| SD | Systolic | 24 hrs | Cat(HvL) | 0 | 0 | 1 | 0 | 0 |
| CoV | Systolic | Day | Cat(HvL) | 0 | 0 | 1 | 0 | 0 |
| CoV | Systolic | 24 hrs | Cat(HvL) | 0 | 0 | 1 | 0 | 0 |
| Night dipping 1 | Wtd average | 24 hrs | Cat(NDvD) | 0 | 0 | 1 | 0 | 0 |
| Night dipping 1 | Systolic | 24 hrs | Cat(NDvD) | 1 | 1 | 2 | 2 | 1 |
| Night dipping 1 | Systolic | 24 hrs | Cat(RvNDvD:NDvD) | 0 | 0 | 1 | 0 | 0 |
| Night dipping 1 | Systolic | 24 hrs | Cat(RvNDvD:RvD) | 0 | 0 | 1 | 0 | 0 |
| Night dipping 1 | Systolic | 24 hrs | Cat(RvNDvDvED:EDvD) | 2 | 1 | 3 | 2 | 1 |
| Night dipping 1 | Systolic | 24 hrs | Cat(RvNDvDvED:EDvND) | 0 | 0 | 0 | 1 | 0 |
| Night dipping 1 | Systolic | 24 hrs | Cat(RvNDvDvED:EDvR) | 0 | 0 | 0 | 1 | 0 |
| Night dipping 1 | Systolic | 24 hrs | Cat(RvNDvDvED:NDvD) | 2 | 1 | 3 | 2 | 1 |
| Night dipping 1 | Systolic | 24 hrs | Cat(RvNDvDvED:RvD) | 2 | 1 | 3 | 2 | 1 |
| Night dipping 1 | Systolic | 24 hrs | Cat(RvNDvDvED:RvND) | 0 | 0 | 0 | 1 | 0 |
| Night dipping 1 | Systolic | 24 hrs | Cat(R v others) | 1 | 0 | 1 | 0 | 1 |
| Pre-awakening 1 | Systolic | 24 hrs | Cat(Quartiles:1v4) | 1 | 0 | 1 | 0 | 0 |
| Pre-awakening 1 | Systolic | 24 hrs | Cat(Quartiles:2v4) | 1 | 0 | 1 | 0 | 0 |
| Pre-awakening 1 | Systolic | 24 hrs | Cat(Quartiles:3v4) | 1 | 0 | 1 | 0 | 0 |
| Sleep trough 1 | Systolic | 24 hrs | Cat(Quartiles:1v4) | 1 | 0 | 1 | 0 | 0 |
| Sleep trough 1 | Systolic | 24 hrs | Cat(Quartiles:2v4) | 1 | 0 | 1 | 0 | 0 |
| Sleep trough 1 | Systolic | 24 hrs | Cat(Quartiles:3v4) | 1 | 0 | 1 | 0 | 0 |
| Sleep trough 2 | Systolic | 24 hrs | Cat(MSvnon-MS) | 0 | 0 | 0 | 1 | 0 |
| **Mixed populations** | | | | | | | | |
| Night dipping 1 | Wtd average | 24 hrs | Cat(NDvD) | 0 | 0 | 1 | 0 | 0 |
| Night dipping 1 | Systolic | 24 hrs | Cat(NDvD) | 2 | 0 | 0 | 0 | 0 |
| Night dipping 2 | Systolic | 24 hrs | Cat(NDvD) | 0 | 0 | 1 | 0 | 0 |
| Night dipping 1 | Systolic | 24 hrs | Cat(RvNDvDvED:EDvD) | 1* | 1* | 1* | 1* | 0 |
| Night dipping 1 | Systolic | 24 hrs | Cat(RvNDvDvED:NDvD) | 1* | 1* | 1* | 1* | 0 |
| Night dipping 1 | Systolic | 24 hrs | Cat(RvNDvDvED:RvD) | 1* | 1* | 1* | 1* | 0 |
| Pre-awakening 1 | Systolic | 24 hrs | Cat(MS v all) | 1* | 1* | 1* | 1* | 0 |
| Pre-awakening 1 | Systolic | 24 hrs | Cat(Quintiles:1v2) | 0 | 0 | 0 | 1 | 0 |
| Pre-awakening 1 | Systolic | 24 hrs | Cat(Quintiles:3v2) | 0 | 0 | 0 | 1 | 0 |
| Pre-awakening 1 | Systolic | 24 hrs | Cat(Quintiles:4v2) | 0 | 0 | 0 | 1 | 0 |
| Pre-awakening 1 | Systolic | 24 hrs | Cat(Quintiles:5v2) | 0 | 0 | 0 | 1 | 0 |
| Sleep trough 2 | Systolic | 24 hrs | Cat(MS v all) | 1* | 1* | 1* | 1* | 0 |
| Sleep trough 2 | Systolic | 24 hrs | Cat(MSvnon-MS) | 0 | 0 | 1 | 0 | 0 |
