# Supplementary material for: Heterogeneity of Prognostic Studies of 24-Hour Blood Pressure Variability: Systematic Review and Meta-Analysis
Source: PLoS One. 2015 May 18;10(5):e0126375. doi: 10.1371/journal.pone.0126375 (PMC4435972; doi:10.1371/journal.pone.0126375)
Supplement: S5 Table — Categorical expressions of relative risks. G—general; M—mixed; H—hypertensive; HD—hypertensive, diabetes; SD—standard deviation; ARV—average real variability; RR—relative risk. Relative risks:>1 increased risk;< 1 reduced risk. (DOCX) [file pone.0126375.s009.docx]

**S5 Table. Comparing predictive power of corresponding systolic and diastolic measures with relative risks scaled per 1 SD increase**

|  |  |  |  |  |  |  | Systolic | | | Diastolic | | |
| --- | --- | --- | --- | --- | --- | --- | --- | --- | --- | --- | --- | --- |
|  | Author | n | Population | BPV measure | BP | Outcome | RR | 95% CI | | RR | 95% CI | |
| 1 | Hansen 2010 | 8938 | G | SD, 24hrs | Systolic | All cause mortality | 1.00 | 0.94 | 1.07 | 1.09 | 1.03 | 1.16 |
| 2 | Mancia 2007 | 2012 | G | SD, 24hrs | Systolic | All cause mortality | 1.00 | 0.88 | 1.14 | 1.10 | 0.97 | 1.24 |
| 3 | Mancia 2007 | 2012 | G | SD, day | Systolic | All cause mortality | 1.13 | 0.99 | 1.29 | 1.23 | 1.08 | 1.40 |
| 4 | Mancia 2007 | 2012 | G | SD, night | Systolic | All cause mortality | 1.06 | 0.94 | 1.21 | 1.13 | 1.01 | 1.27 |
| 5 | Hansen 2010 | 8938 | G | SDdn | Systolic | All cause mortality | 1.08 | 1.01 | 1.15 | 1.16 | 1.09 | 1.23 |
| 6 | Hansen 2010 | 8938 | G | Average real variability | Systolic | All cause mortality | 1.11 | 1.04 | 1.18 | 1.13 | 1.07 | 1.19 |
| 7 | Boggia 2007 | 7458 | M | Night dipping 1 | Systolic | All cause mortality | 1.13 | 1.07 | 1.19 | 1.12 | 1.06 | 1.19 |
| 8 | Muxfeldt 2009 | 556 | H | Night dipping 1 | Systolic | All cause mortality | 1.13 | 0.88 | 1.45 | 0.98 | 0.77 | 1.25 |
| 9 | Hansen 2010 | 8938 | G | SD, 24hrs | Systolic | CV mortality | 1.03 | 0.93 | 1.13 | 1.15 | 1.05 | 1.26 |
| 10 | Mancia 2007 | 2012 | G | SD, 24hrs | Systolic | CV mortality | 0.93 | 0.74 | 1.19 | 1.08 | 0.87 | 1.34 |
| 11 | Mancia 2007 | 2012 | G | SD, day | Systolic | CV mortality | 1.17 | 0.91 | 1.50 | 1.36 | 1.08 | 1.72 |
| 12 | Mancia 2007 | 2012 | G | SD, night | Systolic | CV mortality | 1.04 | 0.83 | 1.29 | 1.14 | 0.93 | 1.41 |
| 13 | Hansen 2010 | 8938 | G | SDdn | Systolic | CV mortality | 1.05 | 0.95 | 1.17 | 1.18 | 1.08 | 1.29 |
| 14 | Hansen 2010 | 8938 | G | Average real variability | Systolic | CV mortality | 1.17 | 1.07 | 1.28 | 1.21 | 1.12 | 1.31 |
| 15 | Boggia 2007 | 7458 | M | Night dipping 1 | Systolic | CV mortality | 1.08 | 0.99 | 1.17 | 1.10 | 1.00 | 1.21 |
| 16 | Muxfeldt 2009 | 556 | H | Night dipping 1 | Systolic | CV mortality | 1.13 | 0.82 | 1.55 | 1.02 | 0.76 | 1.38 |
| 17 | Hansen 2010 | 8938 | G | SD, 24hrs | Systolic | CV events | 1.02 | 0.96 | 1.09 | 1.05 | 0.99 | 1.12 |
| 18 | Eguchi 2009 | 300 | HD | SD, day | Systolic | CV events | 1.15 | 0.83 | 1.58 | 2.03 | 0.88 | 1.35 |
| 19 | Eguchi 2009 | 300 | HD | SD, night | Systolic | CV events | 1.38 | 1.00 | 1.92 | 1.85 | 1.19 | 2.89 |
| 20 | Hansen 2010 | 8938 | G | SDdn | Systolic | CV events | 1.04 | 0.97 | 1.11 | 1.07 | 1.01 | 1.14 |
| 21 | Hansen 2010 | 8938 | G | Average real variability | Systolic | CV events | 1.07 | 1.00 | 1.14 | 1.07 | 1.01 | 1.13 |
| 22 | Pierdomenico 2009* | 1280 | H | Average real variability | Systolic | CV events | 2.07 | 1.31 | 3.28 | 1.36 | 0.92 | 2.02 |
| 23 | Boggia 2007 | 7458 | M | Night dipping 1 | Systolic | CV events | 1.05 | 0.98 | 1.11 | 1.07 | 1.00 | 1.13 |
| 24 | Muxfeldt 2009 | 556 | H | Night dipping 1 | Systolic | CV events | 1.22 | 1.00 | 1.49 | 1.13 | 0.94 | 1.37 |
| 25 | Hansen 2010 | 8938 | G | SD, 24hrs | Systolic | Stroke | 0.98 | 0.88 | 1.09 | 1.08 | 0.99 | 1.99 |
| 26 | Hansen 2010 | 8938 | G | SDdn | Systolic | Stroke | 1.03 | 0.92 | 1.14 | 1.09 | 0.99 | 1.20 |
| 27 | Hansen 2010 | 8938 | G | Average real variability | Systolic | Stroke | 1.10 | 1.00 | 1.21 | 1.14 | 1.05 | 1.23 |
| 28 | Boggia 2007 | 7458 | M | Night dipping 1 | Systolic | Stroke | 1.02 | 0.94 | 1.11 | 1.04 | 0.95 | 1.14 |
| 29 | Muxfeldt 2009 | 556 | H | Night dipping 1 | Systolic | Stroke | 0.98 | 0.71 | 1.34 | 0.96 | 0.72 | 1.28 |
| 30 | Muxfeldt 2009 | 556 | H | Night dipping 1 | Systolic | CHD | 1.24 | 0.91 | 1.70 | 1.11 | 0.83 | 1.48 |
